# The sustainability of public health interventions in schools: a systematic review

**DOI:** 10.1186/s13012-019-0961-8

**Published:** 2020-01-06

**Authors:** Lauren Herlitz, Helen MacIntyre, Tom Osborn, Chris Bonell

**Affiliations:** 1 Department of Public Health, Environments and Society, London School of Hygiene and Tropical Medicine, 15-17 Tavistock Place, London, WC1H 9SH UK; 2 Institute for Health and Human Development, University of East London, Water Lane, London, E15 4LZ UK; 3 Department of Psychology and Human Development, UCL Institute of Education, University College London, 20 Bedford Way, London, WC1H 0AL UK

**Keywords:** Sustainability, Institutionalisation, Organisational change, School health, Systematic review

## Abstract

**Background:**

The sustainability of school-based health interventions after external funds and/or other resources end has been relatively unexplored in comparison to health care. If effective interventions discontinue, new practices cannot reach wider student populations and investment in implementation is wasted. This review asked: What evidence exists about the sustainability of school-based public health interventions? Do schools sustain public health interventions once start-up funds end? What are the barriers and facilitators affecting the sustainability of public health interventions in schools in high-income countries?

**Methods:**

Seven bibliographic databases and 15 websites were searched. References and citations of included studies were searched, and experts and authors were contacted to identify relevant studies. We included reports published from 1996 onwards. References were screened on title/abstract, and those included were screened on full report. We conducted data extraction and appraisal using an existing tool. Extracted data were qualitatively synthesised for common themes, using May’s General Theory of Implementation (2013) as a conceptual framework.

**Results:**

Of the 9677 unique references identified through database searching and other search strategies, 24 studies of 18 interventions were included in the review. No interventions were sustained in their entirety; all had some components that were sustained by some schools or staff, bar one that was completely discontinued. No discernible relationship was found between evidence of effectiveness and sustainability. Key facilitators included commitment/support from senior leaders, staff observing a positive impact on students’ engagement and wellbeing, and staff confidence in delivering health promotion and belief in its value. Important contextual barriers emerged: the norm of prioritising educational outcomes under time and resource constraints, insufficient funding/resources, staff turnover and a lack of ongoing training. Adaptation of the intervention to existing routines and changing contexts appeared to be part of the sustainability process.

**Conclusions:**

Existing evidence suggests that sustainability depends upon schools developing and retaining senior leaders and staff that are knowledgeable, skilled and motivated to continue delivering health promotion through ever-changing circumstances. Evidence of effectiveness did not appear to be an influential factor. However, methodologically stronger primary research, informed by theory, is needed.

**Trial registration:**

The review was registered on PROSPERO: CRD42017076320, Sep. 2017.

Contributions to the literature

- Studies in health care settings have shown that multiple facilitators and barriers affect the sustainability of health interventions beyond effectiveness evaluations and the cessation of funding and/or other resources. This review is the first to apply this evidence-based intervention sustainability in school settings.
- Although we found many commonalities in sustainability factors between education and health care—for example, funding, the work of organisational leaders and staff turnover—we found staff lacked confidence in delivering health promotion without ongoing support and prioritised academic education over health. Perceived effectiveness through witnessing students’ engagement and wellbeing was influential; scientific evidence of effectiveness did not appear to affect sustainability.
- These findings contribute to our understanding of whether, how and why health interventions are sustained, adapted, or discontinued in schools and their ability to have a lasting impact on health outcomes.

## Background

Since the late 1980s, the World Health Organization (WHO) has emphasised schools’ role in promoting health [1, 2]. Increasingly, randomised controlled trials (RCTs) are used to determine the effectiveness of school-based interventions addressing various health outcomes [3–8]. While there has been progress in assessing the effectiveness of such interventions [9–11], and factors affecting implementation [12–14], there is less evidence about sustaining health interventions in schools beyond initial pilots. If effective interventions discontinue, new practices cannot reach wider populations and investments in time, people and resources to initiate and implement them may be wasted [15–18].

Sustainability is a relatively new area of study [19], and most studies come from health care [19, 20]. Conceptual frameworks for sustainability emphasise complexity, whereby practitioners and other actors individually and collectively engage with intervention components and organisational systems to embed, adapt or discard interventions [21–23]. Factors suggested as promoting sustainability include intervention effectiveness, attributes and cost [15, 17, 24]; practitioners’ attributes and activities [21, 24]; the work of intervention champions and organisational leaders [25, 26]; organisational climate and culture; monitoring and evaluation; staff turnover [25, 27]; and the external political and financial climate [26].

While health and education settings may share barriers and facilitators to sustaining new interventions, some factors may differentially affect schools. There may be less political incentive to sustain health interventions; academic education is likely to be prioritised [28–30]. Teachers may need more support and preparation time to deliver curriculums that include health [31] and vary in their commitment to teaching health promotion [13, 31]. Limited interaction between schools and the health sector might impede the identification of funding, resources and training for sustainability [30]. Monitoring ongoing effectiveness might be difficult without routine collection of health data [30].

There has been no systematic review of the sustainability of school-based health interventions. Stirman et al.’s systematic review of research on the sustainability of health interventions found 125 empirical studies published 1980 to 2012 but did not focus on particular settings; only 14 studies assessed school-based interventions [20]. Believing a review of school interventions could prove fruitful, we aimed to examine empirical research on the sustainability of health interventions in schools after start-up funding and/or other resources ceased. As the resources available to schools will likely impact on sustainability, we focus on high-income countries only. The review asks: what evidence exists about the sustainability of school-based health interventions? Do schools sustain public health interventions once start-up funds end? What are the barriers and facilitators affecting the sustainability of public-health interventions in schools in high-income countries?

## Method

### Inclusion/exclusion criteria

A study was included if it:

- Focused on the (dis)continuation of a school-based public-health intervention within the set of schools originally involved in delivering it, and fieldwork was carried out after external funding and/or other resources to implement the intervention had ended
- Used qualitative or quantitative empirical methods
- Was published since 1996 (as these were judged most relevant to current policy contexts) and conducted in an Organisation for Economic Co-operation and Development (OECD) country
- The intervention:

i. Had defined components to be delivered
ii. Targeted children aged 5–18 years
iii. Included health outcomes among its primary outcomes
iv. Focused on obesity/overweight/body size; physical activity/sedentary behaviours; nutrition; tobacco, alcohol/drug use; sexual health; mental health/emotional well-being; violence; bullying; infectious diseases; safety/accident prevention; body-image/eating disorders; skin/sun safety; and oral health [10]
v. Was implemented partly/wholly within school during school hours by teachers, pastoral, managerial or administrative staff, health or wellbeing professionals employed *by the school* or students
vi. Encompassed one or more elements of the Health Promoting Schools (HPS) model [10]: *a formal curriculum*—health education with allocated class time to help students develop the knowledge, attitudes and skills needed for healthy choices; *school ethos or environment*—policies or activities outside the curriculum that promote healthy values and attitudes within school; and/or *family and/or community engagement*—activities engaging families, outside agencies and/or the community

Interventions were excluded if they provided health-information materials only, created new schools or were primarily family/community-based interventions with a minor school component. Interventions which co-located a health service within schools, with services delivered exclusively by clinical providers, were also excluded. The sustainability of such interventions is likely to differ from those delivered partly/wholly by educators or school employees, for example, greater reliance on schools continuing to commission services or the option of service provision at no cost to the school (i.e. through other funding mechanisms), and differences in clinicians and educators’ commitment to sustainability due to differing professional knowledge/roles, peer support and priorities.

### Search strategy

We searched electronic databases for English-language publications between January 1996 and September 2017 (PsycINFO, Social Sciences Citation Index – Social Science & Humanities [Web of Science], British Education Index, PubMed, CINAHL, EMBASE and ERIC). A mixture of free-text and controlled terms was searched in titles/abstracts, and MESH headings where relevant. Synonyms for four concepts were combined: sustainability, school, intervention and public health (see Additional file 1 for full terms used). A comprehensive website search was also carried out (see Additional file 2). School-based studies in Stirman et al.’s review were also screened [20]. The references of included studies were checked, and a citation search was conducted on Google Scholar. Subject-matter experts were contacted to identify unpublished/current research, including authors of included studies (see Additional file 3).

### Screening

All identified studies were imported into the data-management software EPPI-Reviewer 4 [32]. Fifty articles were initially double-screened by two reviewers (LH, HM) on title/abstract: 94% agreement was achieved and discrepancies were discussed to reach a consensus. Reviewers then worked independently, single-screening on title/abstract. Studies were retained if they met the inclusion criteria or if there was insufficient information in the title/abstract to judge. Full-text copies of potentially relevant papers were retrieved and screened independently by the two reviewers to decide on inclusion. If there was uncertainty, studies were discussed by both reviewers (LH, HM) until a consensus was reached, involving a third reviewer (CB) when necessary.

### Data extraction and quality appraisal

We extracted data from each included report on study sample/population; description of the intervention (adapted criteria [33]); key dates, study design/methodology and results for the evaluation of effectiveness (or implementation period for non-evaluated initiatives) and sustainability phase; and information needed for quality appraisal (see Additional file 4). Two reviewers (LH, HM) extracted data from two study reports, comparing their results. Pairs of reviewers (LH, HM or LH, TO) independently completed data extraction for each included report. Differences between reviewers were discussed, including a third reviewer (CB) where necessary.

Two reviewers assessed study reliability using an existing checklist [34]: justification for study focus and methods used; clear aims/objectives; clear description of context, sample and methodology; demonstrated attempts to establishing data reliability and validity; and inclusion of original data. Studies were assigned two ‘weight-of-evidence’ ratings [35], one for reliability and one for relevance to answer the review question, rated ‘low’, ‘medium’ or ‘high’. To achieve ‘high’ reliability, at least five criteria had to be met, for ‘medium’ at least four criteria had to be fully or partially met, and all other studies were rated ‘low’. We also downgraded the reliability of retrospective, cross-sectional studies using self-report data for interventions implemented more than 2 years ago. For a judgement of ‘high’ relevance, studies had to describe, with breadth and depth, factors influencing sustainability and privilege participants’ perspectives (Additional file 5 describes quality criteria and ratings). Studies were not excluded from the synthesis based on their reliability, but greater qualitative weight was given to those assessed as ‘medium’ or ‘high’. The quality-assessment tool was piloted on two studies by each pair of reviewers (LH, HM and LH, TO) with results discussed to ensure consistency. Each included study was then independently quality-assessed by each reviewer with discrepancies discussed, where necessary resolved with a third reviewer (CB).

### Synthesis of results

**Fig. 1:**
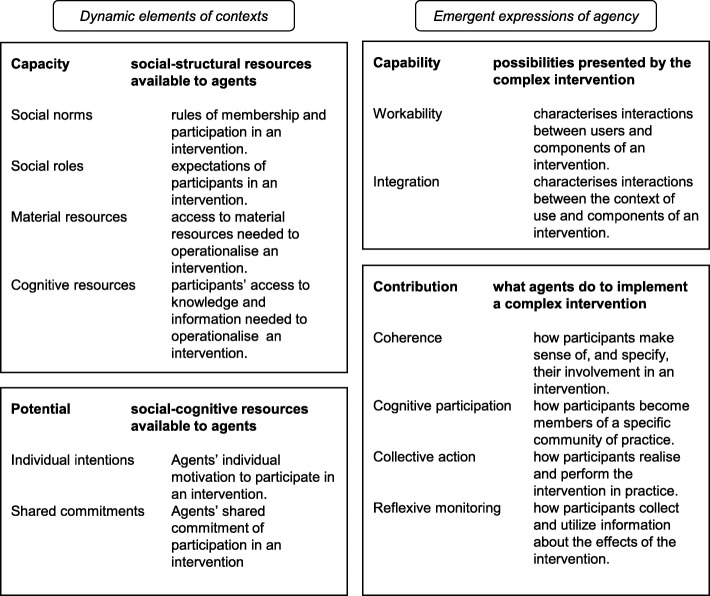
General theory of implementation

We originally intended to use a meta-ethnographic approach as submitted in the protocol [36]. We anticipated finding qualitative studies that were rich in concepts, metaphors and description. However, only one study went beyond description to interpret participants’ views and experiences, and it was not possible to ‘translate’ and synthesise concepts from one study into another. Instead, we conducted thematic synthesis [37] to develop concepts from the mixture of qualitative, quantitative and mixed studies identified. One reviewer (LH) read and re-read studies and carried out line-by-line coding using NVivo 11 software. Inductive codes were developed from the qualitative data (participants’ verbatim quotes and authors’ interpretations) and from authors’ textual reports of quantitative findings. Each code’s data were checked for consistency of interpretation and re-coded as necessary. We used the General Theory of Implementation (GTI [38]) as a sensitising lens; it explains how implementation proceeds over time, building on normalization process theory [21, 39] (Fig. 1 summarises the theory’s constructs). Memos were used to explain codes, their relationships and their alignment with the GTI. GTI informed the overarching structure of themes and sub-themes that was developed. The reliability of each study was checked and referred to as the overall themes were incorporated into a narrative synthesis. The three other reviewers (HM, TO, CB) commented on and discussed a draft of the themes and sub-themes, and a final version was agreed.

This review was registered on PROSPERO (6.9.17, CRD42017076320, [36]) and follows PRISMA reporting standards (Additional File 6).

## Results

**Fig. 2:**
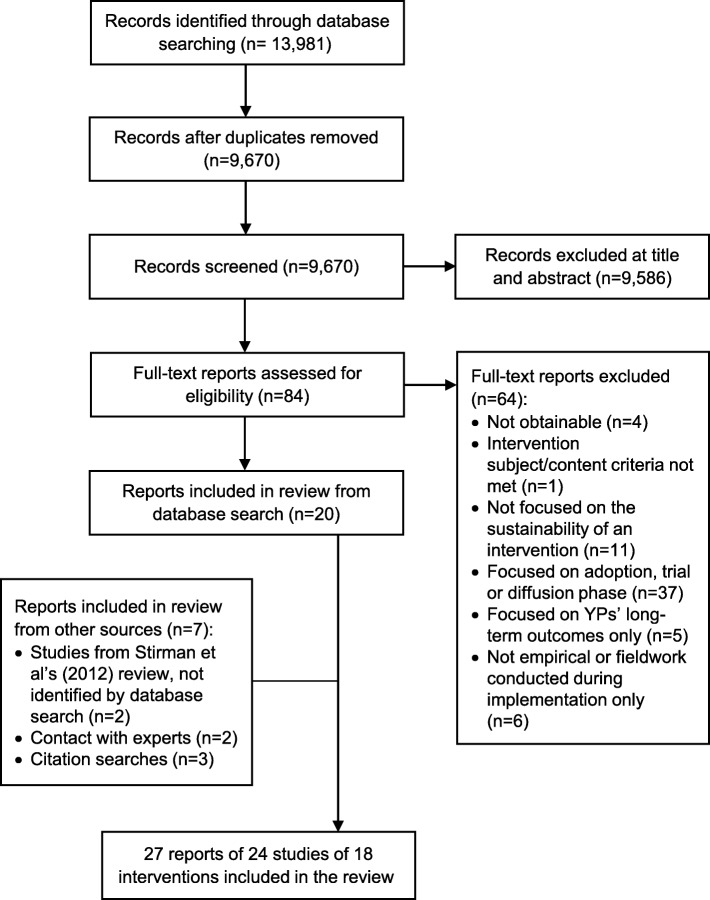
PRISMA flow diagram of study selection process

Of the 9670 unique title/abstracts generated through database-searching (see Fig. 2), we included 20 reports of 19 studies. Other search strategies yielded seven additional reports from five studies. Data extraction was completed for these 24 studies; extraction was not conducted on three doctoral theses [40–42] because each had a corresponding published paper of the same study included in the review [43–45]. In total, the review included 24 studies of 18 different interventions.

### Study characteristics

#### Study origin

Seventeen of the 24 studies were based in the United States (US), of which seven were studies of the Child and Adolescent Trial for Cardiovascular Health (CATCH) intervention [44–60] and the remainder were from Norway [43, 61], the Netherlands [62, 63], Canada [64], England [65] and Germany [66].

#### Intervention characteristics and effectiveness

The largest group of interventions focused on healthy eating and/or physical activity (*n* = 10); the remainder targeted anti-social behaviour (*n* = 2), mental health (*n* = 2), alcohol/drug use (*n* = 2), peer and dating violence (*n* = 1) and workplace health-and-safety (*n* = 1) (see Table 1). Nine were based in elementary/primary schools, eight in middle/high/secondary schools and one in both settings. Intervention length, as initially funded/implemented, ranged from 8 weeks to 3 years (mode = 1 year); three interventions were of unspecified length.

**Table 1:** Description of the interventions in the review

Study #	Intervention name; country; author(s) and year	Health outcome(s) targeted; length of intervention	Country-specific education phase; grade (age); universal or targeted approach	HPS elements			Description of components	Evaluation of effectiveness which preceded assessment of sustainability		Time between effectiveness evaluation and evaluation of sustainability^a^
				Curriculum	Ethos/environment	Family/community		Study design	Evidence of effects on outcomes	
1	*Project Salsa;* United States; Elder et al., 1998 [57]	*Nutrition*; 3 years (for school-based components)	Primary; not stated; universal	X	X	X	• Nutrition education for parents, food service staff, teachers (e.g. health fairs) • Classroom curriculum/learning activities • Links with community institutions • Student advisory committees • Changes to school menus	*Non-experimental pilot evaluation.* 6 intervention schools. Evaluation report was not available.	*Not known.*	2–5 years
2	*Adolescent Suicide Awareness Program (ASAP)*; US; Kalafat and Ryerson 1999 [53]	*Suicidal feelings*; flexible, minimum delivery 3 months	Secondary; grade 10 (15–16 years old); universal	X	X	X	• Classroom curriculum/learning activities • Links with community gatekeepers • Organisational consultation and policies • Educator training • Parent training	*N/A—non-evaluated pilot initiative.*	*Not known.*	5–10 years
3–9	*Child and Adolescent Trial for Cardiovascular Health (CATCH)*; US; Johnson et al. 2003 [52] Kelder et al. 2003 [49] Lytle et al. 2003 [48] McKenzie et al. 2003 [51] Osganian et al. 2003 [55] Parcel et al. 2003 [60] Hoelscher et al. 2004 [56]	*Cardiovascular health*; 3 years	Primary; Grades 3–5 (8–11 years old); universal	X	X	X	• Classroom curriculum/learning activities—changes to PE classes • Classroom curriculum/learning activities—health education lessons • Nutrition programme—changes to school menus, food purchasing and preparation • Family activities and event • No-smoking policy	*cRCT* [67] Schools unit of allocation 56 intervention schools and 40 control	*Effective for primary outcomes* • % of energy intake from total fat in school meals sig. Reduced in intervention schools compared with controls. • Intensity of physical activity (PA) in PE classes increased sig. More in intervention compared with controls. • Dietary knowledge and intentions, and self-reported food choice changes were sig. Greater for intervention schools. • 24-h food recall showed increased total daily energy intake among children in both intervention and control schools with ageing, but increase was greater in control schools. Fat intake was sig. Reduced among children in intervention schools.	5 years
10	*Project ALERT*; US; St Pierre and Kaltreider 2004 [58]	*Substance abuse*; 2 years	Secondary; grades 7 and 8 (12–14 years old); universal	X			• Classroom curriculum/learning activities	*RCT* [68] 8 schools, 6 classes per school randomly assigned to 1 of 3 conditions: experimental groups × 2 and 1 control group.	*No effect on primary outcome, harmful effect for one treatment condition* • No evidence of beneficial effects on substance use. • Harmful effects were found for the teen-assisted intervention condition on marijuana use in the past year, and future expected marijuana use.	< 1 year
11	*School Fruit Programme and the Fruit and Vegetables Make the Marks (FVMM)*; Norway; Bere 2006 [61]	*Fruit and vegetable consumption*; 1 year	Primary; grade 6 (11 years old); universal	X	X	X	• Subscription to the national fruit and vegetable programme (free in trial phase) • Classroom curriculum/learning activities • Parent newsletters	*RCT* [61] 9 intervention schools, 10 control schools.	*Effective for primary outcome* • Strong intervention effects were observed for fruit and vegetables (F&V) eaten at school and all day. • Average F&V intake was 0.6 portions higher in the intervention group than controls at school & all day.	1 year
12	*Untitled - intervention focused on water consumption*; Germany; Muckelbauer et al. 2009 [66]	*Overweight*; 2 years	Primary; grades 2 and 3 (7–9 years old); universal	X	X		• Installation of school water fountain • Classroom curriculum/learning activities	*cRCT* [69] City unit of allocation 17 intervention schools, 16 control schools.	*Effective for some but not all primary outcomes* • The risk of being overweight was sig. Reduced in the intervention group compared with controls. • No sig. Differences for BMI. There was no general weight-reducing effect. • Changes in water consumption higher in the intervention group compared with controls. No effects on juice or soft drink consumption.	< 1 year
13	*European Network of Health-Promoting Schools*; Norway; Tjomsland et al. 2009 [43]	*Healthy lifestyles*; 3 years	Primary and secondary; grades 5–10 (10–16 years old); universal	X	X	X	• Health integrated into school policies • Needs assessment • A variety of activities e.g. curriculum, meals, school environment, parent-involvement (differed by school) • National, regional, and international conferences	*Non-experimental pilot evaluation* 10 intervention schools. Evaluation report on outcomes not available.	*Not known.*	9 years
14	*Winning with Wellness*; US; Schetzina et al. 2009 [50]	*Nutrition, physical activity, obesity*; 1 year	Primary; grades 3 and 4 (8–10 years old) universal	X	X		• 5 min desk-side exercises • 2 x classroom curriculum—nutrition and health education • Changes to school menus and vending machines. • Snack preparation demonstrations • Walking trails • School health services • Health promotion for staff	*Non-experimental pilot evaluation* [50] 1 school	*Effective for some but not all primary outcomes* • No sig. Changes in BMI. • Students were sig. More active at school after intervention implementation than before, with an increase of approx. 886 steps per day. • Sig. fewer unhealthy foods were being offered & purchased/served to students after implementation than before.	< 1 year
15	*First Step to Success*; US; Loman et al. 2010 [59]	*Anti-social behaviour*; 18 months	Primary; grades K to 2 (5–8 years old); targeted		X	X	• Universal screening • Consultant-based behavioural intervention with teacher, child and peers • Parent training	*Non-randomised controlled trial* [70] No. of schools not stated.	*Effective for primary outcome* • Sig. pre-post behavioural changes—adaptive, aggression, maladaptive, academic engaged time—for the intervention group. • No sig. Difference in teachers’ perception of how positively or negatively other children in the class viewed the target child.	4–10 years
16	*GreatFun2Run*; England; Gorely et al. 2011 [65]	*Physical activity and fruit and vegetable consumption*; 10 months	Primary; grade not stated (7–11 years old); universal	X	X	X	• Classroom curriculum/learning activities • Participation in two running events • An interactive website • A local media campaign	*Non-randomised controlled trial* [71] 4 intervention schools, 4 control schools	*Effective for some but not all primary outcomes* • Sig. increase in students’ daily steps & total time in MVPA in intervention compared to control schools. • Older participants in intervention schools showed a sig. Slowing in the rate of increase in estimated % body fat, BMI, & waistline. • No difference between groups in F&V consumption, aerobic fitness, knowledge of healthy lifestyles, perceived competence, enjoyment of PA, or intrinsic motivation.	1 year 9 months
17	*Fourth R program*; Canada; Crooks et al. 2013 [64]	*Peer and dating violence*; 1 year	Secondary; grade 9 (14–15 years old); universal	X		X	• Classroom curriculum/learning activities • Parent newsletters	*cRCT* [72] Schools unit of allocation 10 intervention schools, 10 control schools	*Effective for some but not all primary outcomes* • Physical dating violence (PDV) was sig. Higher for students in control schools than for those in intervention schools. • Boys in intervention schools were less likely than boys in control schools to engage in dating violence. However, girls had similar rates of PDV in both groups. • Differences between control & intervention groups were not sig. For physical peer violence, substance use, or condom use.	2 or more years, range not stated.
18	*New Moves*; US; Friend et al. 2014 [47]	*Obesity, physical activity, eating behaviours, body image*; 1 year	Secondary; grade not stated (14–16 years old); targeted	X	X	X	• 3 x classroom curriculum/learning activities—all-girls physical education class, nutrition, and social support • Individual counselling sessions • Lunch get-togethers • Parent postcards and event	*cRCT* [73] Schools unit of allocation 6 intervention schools, 6 control schools	*Effective for some but not all primary outcomes* • Sig. differences between intervention & control students in changes in: stage of change for PA, goal setting for PA and self-efficacy to overcome barriers to PA; total non-sedentary activity; stage of change for F&V, & goal setting for healthy eating; portion control; unhealthy weight control behaviours; body satisfaction; athletic competence & self-worth. • Changes were non-significant in: body fat & BMI, total PA and MVPA, TV time, & stage of change TV, F&V intake & sugar-sweetened beverages, and breakfast, binge eating, appearance	1–2 years
19	*Youth@work: Talking Safety*; US; Rauscher et al. 2015 [54]	*Workplace safety and health*; not specified—6 sessions.	Secondary; grade not stated (age not stated); universal	X			• Classroom curriculum/learning activities	*Non-experimental pilot evaluation.* Evaluation report was not available.	*Not known*	1–9 years
20	*Cognitive Behavioral Intervention for Trauma in Schools (CBITS)*; US; Nadeem and Ringle 2016 [46]	*Post-traumatic stress disorder, anxiety and depression*; 1 year	Secondary; grade 6 (11 years old); targeted		X	X	• 10 group sessions • 1–3 individual sessions • Parent and teacher education	*Non-experimental pilot evaluation* [74] 30 intervention schools.	*Effective for primary outcome* • There was a sig. Pre- to post-intervention decline in PTSD symptoms.	2 years
21	*Good Behavior Game (GBG)*; The Netherlands; Dijkman et al. 2017 [63]	*Anti-social behaviour*; 1 year	Primary; grade 2 (6–7 years old); universal		X		• Behavioural approach in classroom	*N/A—non-evaluated pilot initiative*	*Not known*	1 year 9 months
22	*TAKE 10!*; US; Goh et al. 2017 [44]	*Physical activity and on-task behaviour*; 8 weeks	Primary; grades 3–5 (8–11 years old); universal		X		• Classroom activity	*Non-experimental pilot evaluation* [42, 85] 1 intervention school.	*Effective for some but not all primary outcomes* • No sig. Effect on mean daily in-school steps. • No sig. Effect on average daily in-school moderate intensity PA levels of students. • Sig. effect on MVPA levels and vigorous intensity PA. • There was a mean % decrease of on-task behaviour by 7.7% during the baseline period & a mean percentage increase of on-task behaviour by 7.2% during the intervention period.	< 1 year
23	*School outdoor smoking ban*; The Netherlands; Rozema et al. 2018 [62]	*Tobacco use*; unspecified/continuous	Secondary; grades n/a (12–18 years old); universal		X		• Smoking ban everywhere on school grounds for everyone	*N/A—non-evaluated pilot initiative*	*Not known*	1–40 years However, 64% of schools had implemented the ban in the last 3 years.
24	*Health Optimizing PE (HOPE)*; US; Egan et al. 2019 [45]	*Physical activity*; 2 years	Secondary (middle); grades 6–7 (11–13 years old); universal	X	X	X	• Provision of technology resources • Before and after school activities • Classroom curriculum/learning activities • Family event • Parent education event	*Non-experimental pilot evaluation* [86] 1 intervention school.	*Effective for primary outcome* • Sig. difference between baseline & end of year 2 for various fitness activities & amount of PA time in class. • There was a sig. Improvement on test of knowledge of PA and healthy eating between baseline & year 1, & baseline & year 2. • The mean number of MVPA minutes (daily) declined steadily over the course of the study.	< 1 year

^a^Estimated as the time between the last year of the effectiveness evaluation (or the end of the implementation period for non-evaluated initiatives) and the last year of the sustainability phase evaluation

During initial implementation in schools prior to assessing sustainability, effectiveness evaluations were conducted of 15 interventions; three were not evaluated [53, 62, 63], though one [63] had been assessed by RCT in other schools [75] (see Table 1). Of the effectiveness evaluations, six interventions (relating to 12 studies) were assessed by RCTs [47–49, 51, 52, 55, 56, 58, 60, 61, 64, 66], two by using non-randomised controlled studies [59, 65] and seven by uncontrolled evaluations [43–46, 50, 54, 57]; evaluation reports were inaccessible for three interventions). Of the 12 interventions for which evaluation reports were available, five interventions were effective for all primary outcomes, six interventions were effective for some but not all primary outcomes and one intervention had no effect and a negative effect for one treatment condition (see Table 1).

#### Study design/methods

Ten studies of sustainability used quantitative cross-sectional designs (42%) [50–54, 56, 59, 60, 64, 66], and one study employed a quantitative longitudinal design [61] (see Table 2). All except one of these used questionnaires to examine sustainability. Six studies employed qualitative designs [43–46, 48, 58]. Seven studies used mixed-methods [47, 49, 55, 57, 62, 63, 65]. Ten studies (42%) used a comparison group of schools [47–49, 51–53, 55, 56, 61, 65].

**Table 2:** Sustainability study design and weight of evidence ratings of the intervention

Study #	Intervention; author(s) and year	Study design	Methods	No. of former intervention (FI) and comparison group (CG) schools; response rates	Reporting on sustainability	W1—reliability	W2—relevance
1	*Project Salsa*; Elder et al. 1998 [57]	• *Mixed-methods.* • Unknown whether data collected at single or multiple time points. • No comparison group.	Focus groups, questionnaires, oral feedback.	6 FI schools; 100% (implied)	School-level	Low	Low
2	*Adolescent Suicide Awareness Program (ASAP)*; Kalafat and Ryerson 1999 [53]	• *Quantitative, cross-sectional.* • Data collected at single time point. • Comparison group for survey—another suicide prevention intervention, no comparison group for interviews.	Survey of all public high schools in one county, plus structured interviews with a sub-sample of schools.	24 FI schools; 73% 7 CG schools; 54%	School-level	Low	Med
3	*Child and Adolescent Trial for Cardiovascular Health (CATCH) – health education curriculum*; Johnson et al. 2003 [52]	• *Quantitative, cross-sectional.* • Data collected at single time point. • Two comparison groups—former control schools who received a low dose of the intervention at the end of the trial phase and an unexposed comparison group who received no intervention.	Questionnaires.	56 FI schools; 100% 20 CG1^a^ schools; 12 CG2^b^ schools; 100%.	Staff-level	High	Low
4	*CATCH – PE component*; Kelder et al. 2003 [49]	• *Mixed-methods, cross-sectional*. • Data collected at single time point. • Two comparison groups—former control schools who received a low dose of the intervention at the end of the trial phase and an unexposed comparison group who received no intervention.	Questionnaires, observation of PE lessons, in-depth interviews.	56 FI schools; 100% 20 CG1 schools; 12 CG2 schools; 100%	Staff-level	Med	Med
5	*CATCH – all components*; Lytle et al. 2003 [48]	• *Qualitative, cross-sectional.* • Data collected at a single time point. • One comparison group—former control schools.	Interviews.	56 FI schools; 100% 20 CG1 schools; 100%	Staff-level	Med	High
6	*CATCH – PE component*; McKenzie et al. 2003 [51]	• *Quantitative, cross-sectional.* • Data collected at a single time point. • One comparison group—former control schools.	Observation of PE lessons, questionnaires.	56 FI schools; 100% 20 CG1 schools; 100%	Staff-level	Low	Low
7	*CATCH – food service component*; Osganian et al. 2003 [55]	• *Mixed-methods, cross-sectional.* • Data collected at a single time point. • One comparison group— former control schools.	Monitoring data, interviews and questionnaires.	56 FI schools; 100% 20 CG1 schools; 100%	School-level and staff-level	High	Med
8	*CATCH – school climate*; Parcel et al. 2003 [60]	• *Quantitative, cross-sectional.* • Data collected at single time point. • No comparison group.	Questionnaires, observation of PE lessons, monitoring data.	56 FI schools; 100%	School-level	High	Low
9	*CATCH – all components*; Hoelscher et al. 2004 [56]	• *Quantitative, cross-sectional.* • Data collected at single time point. • Two comparison groups—former control schools who received a low dose of the intervention at the end of the trial phase and an unexposed comparison group who received no intervention.	Questionnaires, observation of PE lessons, monitoring data.	56 FI schools; 100% 20 CG1 schools; 12 CG2 schools; 100%	School-level and staff-level	High	Low
10	*Project ALERT*; St Pierre and Kaltreider 2004 [58]	• *Qualitative.* • Data collected at single time point. • No comparison group.	Interviews.	8 FI schools; 100%	School-level	Low	Low
11	*School Fruit Programme and the Fruit and Vegetables Make the Marks (FVMM)*; Bere 2006 [61]	• *Quantitative, longitudinal.* • Data collected over multiple time points, following the students’ outcomes over time (same individuals). • Comparison group.	Questionnaires.	9 FI schools; 100% 10 CG schools; 100%	School-level	High	Low
12	*Untitled - intervention focused on water consumption*; Muckelbauer et al. [66]	• *Quantitative, cross-sectional*. • Data collected at multiple time points (not necessarily the same individuals). • No comparison group.	Questionnaire, (structured) telephone interview, measure water flow of fountains.	17 FI schools; 100%	School-level	Med	Low
13	*European Network of Health-Promoting Schools*; Tjomsland et al. 2009 [43]	• *Qualitative*. • Data collected at single time point. • No comparison group.	Telephone interviews and document analysis.	7 FI schools; 70%	School-level	Med	High
14	*Winning with Wellness*; Schetzina et al. 2009 [50]	• *Quantitative, cross-sectional.* • Data collected at multiple time points (not necessarily the same individuals). • No comparison group.	Survey.	1 FI school; 100%	Staff-level	Med	Low
15	*First Step to Success (FSS)*; Loman et al. 2010 [59]	• *Quantitative, cross-sectional.* • Data collected at a single time point. • No comparison group.	Structured interview by telephone or in-person and website process evaluation tool.	29 FI schools; 13/29 school districts (45%) had continued to use the intervention. District administrators nominated schools.	School-district level and school-level	Low	Low
16	*GreatFun2Run*; Gorely et al. 2011 [65]	• *Mixed-methods, cross-sectional and longitudinal*. • Data on students’ outcomes collected over multiple time points (same individuals). • Data on teachers and students’ views of the intervention collected at a single time point. • Comparison group used for student outcomes	Observation, anthropometric measures, focus groups, interviews.	4 FI schools; 100%	Staff-level	High	Med
17	*Fourth R program*; Crooks et al. 2013 [64]	• *Quantitative cross-sectional*. • Study sample were teachers trained in the intervention two or more years ago. • Data collected at single time point. • No comparison group.	Online survey.	Not known	Staff-level	Low	Med
18	*New Moves*; Friend et al. 2014 [47]	• *Mixed-methods, cross-sectional*. • Data collected at single time point. • Comparison group—teachers received a lower dose of New Moves at the end of the trial.	Questionnaire, interviews and PE lesson observation.	6 FI schools; 100% 6 CG schools; 100%	School-level	Med	Med
19	*Youth@work: Talking Safety*; Rauscher et al. 2015 [54]	• *Quantitative, cross-sectional*. • Study sample were teachers that were trained in the intervention between 2004 and 2012. • Data collected at single time point. • No comparison group.	Telephone survey.	Not known	Staff-level (sustainability score)	Low	Low
20	*Cognitive Behavioral Intervention for Trauma in Schools (CBITS)*; Nadeem and Ringle 2016 [46]	• *Qualitative*. • Study sample were clinicians who had worked in former intervention schools. • Data collected at single time point. • No comparison group.	Interviews.	Not known	Staff-level	High	High
21	*Good Behavior Game (GBG)*; Dijkman et al. 2017 [63]	• *Mixed-methods, cross-sectional*. • Data collected at single time point. • No comparison group.	Questionnaire and interviews.	16 FI schools; 94%	School-level (sustainability score)	Med	High
22	*TAKE 10!*; Goh et al. 2017 [44]	• *Qualitative*. • Data collected at single time point. • No comparison group.	Interviews.	2 FI schools; opportunity sample.	Staff-level	Med	Med
23	*School outdoor smoking ban*; Rozema et al. 2018 [62]	• *Mixed-methods, cross-sectional*. • No comparison group.	Questionnaire for all secondary schools enquiring about use of outdoor smoking ban. Additional questionnaire for those with ban. Qualitative interviews with sub-sample of schools conducted 6 months later.	438 schools; response rate not known—schools currently with the intervention.	School-level (sustainability score)	Low	Med
24	*Health Optimizing PE (HOPE)*; Egan et al. 2019 [45]	• *Qualitative single case study.* • Data collected at multiple time points from the research team—interviewed twice during the trial phase, and once 1 year post-trial phase. • Data collected at single time point from teachers and students. • No comparison group.	Document analysis, interviews, focus group.	1 FI school; 100%	School-level	High	Med

^a^CG1—20 schools who received a lower dose of CATCH at the end of the trial. ^b^CG2—12 schools who did not receive the intervention

#### Timeframe examined

Timeframes between the effectiveness evaluation (or implementation period in non-evaluated initiatives) and the study of sustainability varied (Table 1). Five studies examined sustainability less than a year after the effectiveness evaluation [44, 45, 50, 58, 66]. Four were conducted 1 to 2 years later [47, 61, 63, 65]; ten took place 2 to 5 years after the evaluation [47, 49, 50, 52, 53, 56–58, 61, 65] and five examined sustainability more than 5 years later [43, 53, 54, 59, 62].

#### Study participants

Six studies sampled several classroom teachers per school [44, 45, 50, 52, 64, 65], and six of the CATCH studies sampled multiple staff members and/or school-district level personnel per school [48, 49, 51, 55, 56, 60] (see Additional file 7). Three studies sampled school principals only [43, 62, 66], four sampled one teacher or staff-member per school [47, 54, 59, 63] and one sampled clinicians delivering the intervention plus school-district level personnel [46]. Three collected data from students [45, 61, 65], and one interviewed the research team implementing the intervention [45]. Three studies provided no details on staff-level participants [53, 57, 58].

#### Study quality

Study reliability and relevance varied. On reliability, seven studies were rated high, nine medium and eight low. On relevance for answering the review question, four studies were rated high, ten medium and ten low. Only one study was rated high on relevance and reliability [46] (see Table 2).

#### Explicit use of conceptual framework

Most studies did not use a conceptual theory/framework. Of those that did (*n* = 9), a variety of sustainability [17, 76–79] and implementation frameworks [80–82] were used. Only one study [43] drew on conceptual frameworks specific to educational settings [83].

#### Reporting of sustainability

Eleven studies reported on intervention sustainability at school-level [43, 45, 47, 53, 57, 58, 60–63, 66], ten at staff-level [44, 46, 48–52, 54, 64, 65], two at the school- and staff-level [55, 56] and one at school-district and school-level [59] (Table 2). Seventy-six percent of studies with a curriculum component [45, 47–53, 56–58, 64, 65], 67% of studies with a school-environment component [43–47, 53, 55, 57, 61, 66] and one third of studies containing a family/community component reported on its sustainability [45, 46, 48, 53] (see Table 3). Around half of studies (46%) of multi-component interventions reported sustainability of some but not all components.

**Table 3:** Summary of results on the sustainability of the intervention

Study #	Intervention; author(s) and year	Sustainability of the intervention (FI = former intervention, CG = comparison group)		
		Curriculum	Ethos/environment	Family/community
1	*Project Salsa*; Elder et al. 1998 [57]	One school (17%) continued nutrition-related activities for students and parents.	No schools continued student advisory committees and changes to school menus. Nutrition education classes for adults continued, unknown if this occurred in all schools.	The nutrition information provided by a community institution was discontinued and replaced with a different intervention, delivered by parent volunteers.
2	*Adolescent Suicide Awareness Program (ASAP)*; Kalafat and Ryerson 1999 [53]	96% of FI schools continued student training, although at a lesser dosage, compared to 100% of CG schools.	67% of schools had written policies and procedures for responding to at-risk students, compared to 86% of CG schools. 8% of schools continued educator training, compared to 0% of CG schools.	All schools retained links with community agencies. 13% of schools continued parent training compared to 0% of CG schools.
3	*Child and Adolescent Trial for Cardiovascular Health (CATCH) – health education curriculum*; Johnson et al. 2003 [52]	19% of teachers in FI schools used CATCH health education activities, compared to 5% in CG1^a^ schools and 0% in CG2^b^ schools. 23% of teachers in FI schools used CATCH health education materials, compared to 11% in CG1 schools and 0% in CG2 schools. 69% of teachers in FI schools taught zero hours of CATCH in the current school year, compared to 84% in CG1 schools, and 99% in CG2 schools.		
4	*CATCH – PE component*; Kelder et al. 2003 [49]	35% of teachers in FI schools had CATCH PE materials available, compared to 19% in CG1^a^ schools. 32% of teachers in FI schools had used CATCH PE materials, compared to 22% in CG1 schools. There were no sig. differences between study groups (FI, CG1, or CG2^b^) in the amount of physical activity.		
5	*CATCH – all components*; Lytle et al. 2003 [48]	34% of staff from FI schools said they were partially implementing the health education curriculum, compared to 23% of staff from CG1^a^ schools. 66% said it was *not* implemented their school, compared to 62% in CG1 schools. 24% of staff from FI schools said they were still implementing CATCH PE. 70% of staff from FI schools said they used elements of it, compared to 93% from CG1 schools. 6% of staff from FI schools said they had discontinued CATCH PE, compared to 7% of staff from CG1 schools.	None of the food service staff from FI schools said they were fully implementing the food service component ‘Eat Smart (ES)’. 27% of the respondents from CG1 schools said ES was not being used at their school. Most district-level respondents said that some of the ES guidelines were being followed. Sustainability of the no-smoking policy not reported.	4% of staff from FI schools said they carried out some parts of the family component. All other staff indicated it had been discontinued.
6	*CATCH – PE component*; McKenzie et al. 2003 [51]	70% of teachers from FI schools who had had CATCH PE training reported using the CATCH PE curriculum, compared to 57% from CG1^a^ schools. There were no sig. differences between FI and CG1 schools in the amount of physical activity in PE lessons and class energy expenditure.		
7	*CATCH – food service component*; Osganian et al. 2003 [55]		25% of cooks in FI schools said the ES manual was present in the school kitchen compared to 15% in CG1^a^ schools. 15% of cooks in FI schools said they used it compared to 3% in CG1 schools. 34% of cooks in FI schools said the recipe box was present in the kitchen compared to 20% in CG1 schools 32% of cooks in FI schools said they used it compared to 12% in CG1 schools.	
8	*CATCH – school climate*; Parcel et al. 2003 [60]		Schools in which principals and teachers were more open were sig. more likely to be teaching more hours of CATCH. ‘Open’ principals were supportive, low on rigid monitoring/control and low on restrictiveness. ‘Open’ teachers were highly collegial, had a network of social support and were engaged with school. Schools in which principals and teacher were more open, and schools higher in organisational health, were sig. more likely to have a greater percentage of calories from saturated fat in school lunches.	
9	*CATCH – all components*; Hoelscher et al. 2004 [56]	No differences between study groups (FI, CG1^a^, CG2^b^) and % of class time spent in moderate to vigorous physical activity or vigorous physical activity. All study groups exceeded the CATCH goal of 90 min of PE/week. Teachers reported teaching only about two CATCH lessons during the previous school year, a much lower dosage than the original intervention. Over 88% of PE teachers and 60% of classroom teachers reported using the CATCH PE activity box in the previous school year.	30% of FI schools achieved the total fat goal of < 30%, compared to 10% of CG1 schools and 17% CG2 schools. 45% of FI schools achieved the saturated fat goal of < 10%, compared to 30% of CG1 schools and 17% of CG2 schools. Most ES guidelines implemented consistently across all study conditions. No schools met the ES guidelines for sodium. Sustainability of the no-smoking policy was not reported.	The family component was taught infrequently.
10	*Project ALERT*; St Pierre and Kaltreider 2004 [58]	38% of schools continued the curriculum.		
11	*School Fruit Programme and the Fruit and Vegetables Make the Marks (FVMM)*; Bere 2006 [61]	Sustainability of the classroom curriculum/learning activities was not reported.	44% of schools continued to participate in the School Fruit Programme (SFP) (paying for it), compared to 30% of CG schools (*n* = 3). 66% of students subscribed to the School Fruit Programme, compared to 21% of students in CG schools. Students from FI schools who continued to participate in the SFP ate 0.4 portions more FV at school than students from FI schools that discontinued participation.	Sustainability of the parent newsletters was not reported.
12	*Untitled - intervention focused on water consumption*; Muckelbauer et al. [66]	Sustainability of the classroom curriculum/learning activities was not reported.	65% of schools retained the water fountain. The mean water flow was highest in the first 3 months of implementation. Afterwards, it decreased by about 35% until the end of the intervention, and remained stable between implementation and sustainability phases.	
13	*European Network of Health-Promoting Schools*; Tjomsland et al. 2009 [43]	Sustainability of specific classroom curriculum/learning activities was not reported.	86% of schools had sustained and developed health promotion practices—specific activities and policies were not reported. 71% of schools referred to aspects of health promotion in their vision statements/priority areas. Sustainability of the needs assessment and national, regional and international conferences were not reported.	Sustainability of specific family/community activities was not reported.
14	*Winning with Wellness*; Schetzina et al. 2009 [50]	50% of teachers reported teaching students the nutrition curriculum. Sustainability of the health education curriculum was not reported.	100% of teachers reported using the 5 min desk-side exercises. Sustainability of the changes to school menus and vending machines, snack preparation demonstrations, use of walking trails, school health services and health promotion activities for staff was not reported.	
15	*First Step to Success (FSS)*; Loman et al. 2010 [59]		8/13 school districts (62%) reported at least one school was continuing to use the behavioural intervention. 72% of the schools nominated by district administrators reported sustainment (mean duration was 7.1 years). 28% of the schools had discontinued implementation (mean duration was 2.4 years).	Sustainability of the parent-training component was not reported.
16	*GreatFun2Run*; Gorely et al. 2011 [65]	25% of teachers were currently using any of the intervention resources. There were no sig. differences between students from FI and CG schools in steps per day or moderate to vigorous physical activity at the time of the sustainability study (in contrast to trial phase).	The sustainability of the use of the summer activity wall planner and website was not reported.	The sustainability of the running events was not reported.
17	*Fourth R program*; Crooks et al. 2013 [64]	72% of teachers said they had implemented the intervention in the most recent school year. During the most recent year of implementation: 40% said they had implemented 81% or more of the programme; 25% said 61–80% of the programme; 18% said 41–60% of the programme; 13% said 21–40% of the programme; 5% said less than 20% of the programme		The sustainability of the parent newsletters was not reported.
18	*New Moves*; Friend et al. 2014 [47]	83% of schools continued the intervention to some degree. One school closed; one discontinued the intervention. Of schools that remained open (*n* = 11): • 91% offered an all-girls PE class 4 times a week. In 9/10 observed classes, most girls met the goal for being active at least 50% of the class. • 45% of schools continued to implement nutrition and social support classes.	27% of schools offered individual coaching sessions, though less frequently than the intervention specified. 0% of schools continued lunch get-togethers.	Sustainability of the parent postcards and event were not reported.
19	*Youth@work: Talking Safety*; Rauscher et al. 2015 [54]	81% of teachers had taught the curriculum more than once since being trained in it, with a mean sustainability score of 10.1 (SD = 6.6, maximum score 18). The mean fidelity score was 2.1 (SD 2.2, maximum score 6).		
20	*Cognitive Behavioral Intervention for Trauma in Schools (CBITS)*; Nadeem and Ringle 2016 [46]		50% of clinicians implemented the counselling intervention 1 year after the trial phase. 0% of clinicians implemented the intervention 2 years after the trial phase.	Sustainability of parent outreach activities not reported.
21	*Good Behavior Game (GBG)*; Dijkman et al. 2017 [63]		The mean sustainability score was 8.7 (range 2–14, maximum score 20).	
22	*TAKE 10!*; Goh et al. 2017 [44]		20% of teachers implemented the activities regularly (2 or more times a week; during the trial phase, teachers implemented the intervention on average once a day). Some teachers (numbers not given) implemented it less regularly (once a week or less). A few teachers (numbers not given) discontinued the intervention.	
23	*School outdoor smoking ban*; Rozema et al. 2018 [62]		The mean sustainability score was 5.70 (SD 0.9, maximum score 7).	
24	*Health Optimizing PE (HOPE)*; Egan et al. 2019 [45]	Teachers (numbers not given) were still using the technology resources. The classroom curriculum was discontinued.	One element of the before and after school activities—‘Intramurals’ was discontinued and then reinstated 2 months later. Another before and after school activity was discontinued.	The family fun run event continued (the event had existed pre-trial phase). The parent education event was discontinued.

^a^CG1—20 schools who received a lower dose of CATCH at the end of the trial. ^b^CG2—12 schools who did not receive the intervention

#### Sustainability of the interventions

No interventions were entirely sustained; Table 3 summarises the percentage of staff or schools sustaining each component. Studies were heterogeneous: all interventions had some components that were continued by some schools or staff, except for one intervention that was completely discontinued two years after the effectiveness evaluation [46]. There were no noticeable patterns between evidence of effectiveness during implementation and sustainability, unaided by inconsistency and gaps in the reporting of sustainability and evidence of effectiveness (see Table 4).

**Table 4:** Effectiveness and sustainability

Study #	Intervention name; author(s) and year	Effects on outcome(s) summarised	% of schools/staff that sustained the curriculum component	% of schools/staff that sustained the ethos/environment component	% of schools/staff that sustained the family component
3–9	*Child and Adolescent Trial for Cardiovascular Health (CATCH)*; Johnson et al. 2003 [52]; Kelder et al. 2003 [49]; Lytle et al. 2003 [48]; McKenzie et al. 2003 [51]; Osganian et al. 2003 [55]; Parcel et al. 2003 [60]; Hoelscher et al. 2004 [56]	Effective for primary outcomes	23% of teachers had used health education materials 32% of teachers had used PE materials 88% of PE specialists had used PE materials	15% of cooks said they used the intervention manual. 32% of cooks said they used the intervention recipe box.	4% of staff
11	*School Fruit Programme and the Fruit and Vegetables Make the Marks (FVMM)*; Bere 2006 [61]	Effective for primary outcomes	Not reported	44% of schools	Not reported
15	*First Step to Success*; Loman et al. 2010 [59]	Effective for primary outcomes	n/a	Not reported	Not reported
20	*Cognitive Behavioral Intervention for Trauma in Schools (CBITS)*; Nadeem and Ringle 2016 [46]	Effective for primary outcomes	n/a	0% of clinicians	0% of teachers
24	*Health Optimizing PE (HOPE)*; Egan et al 2019 [45]	Effective for primary outcomes	0% of schools (NB one school in study)	One activity continued, one activity discontinued	0% of teachers
12	*Untitled - intervention focused on water consumption*; Muckelbauer et al. 2009 [66]	Effective for some but not all primary outcomes	Not reported	65% of schools	n/a
14	*Winning with Wellness*; Schetzina et al. 2009 [50]	Effective for some but not all primary outcomes	50% of teachers (not all classroom activities reported)	Not reported	n/a
16	*GreatFun2Run*; Gorely et al. 2011 [65]	Effective for some but not all primary outcomes	25% of teachers	Not reported	Not reported
17	*Fourth R program*; Crooks et al. 2013 [64]	Effective for some but not all primary outcomes	72% of teachers	n/a	Not reported
18	*New Moves*; Friend et al. 2014 [47]	Effective for some but not all primary outcomes	91% of schools continued PE; 45% continued health education	27% of schools continued individual staff-student coaching sessions; 0% of schools staff-student lunch get-togethers	Not reported
22	*TAKE 10!*; Goh et al. 2017 [44]	Effective for some but not all primary outcomes	n/a	20% of teachers	n/a
10	*Project ALERT*; St Pierre and Kaltreider 2004 [58]	No effect on primary outcome, harmful effect for one treatment condition	38% of schools	n/a	n/a
1	*Project Salsa;* Elder et al., 1998 [57]	n/k	17% of schools	0% of schools	Not reported
2	*Adolescent Suicide Awareness Program (ASAP)*; Kalafat and Ryerson 1999 [53]	n/k	96% of schools	67% of schools	13% of schools
13	*European Network of Health-Promoting Schools*; Tjomsland et al. 2009 [43]	n/k	Not reported	71% of schools	Not reported
19	*Youth@work: Talking Safety*; Rauscher et al. 2015 [54]	n/k	Not reported	n/a	n/a
21	*Good Behavior Game (GBG)*; Dijkman et al. 2017 [63]	n/k	n/a	Not reported	n/a
23	*School outdoor smoking ban*; Rozema et al. 2018 [62]	n/k	n/a	Not reported	n/a

## Thematic synthesis of barriers and facilitators of sustainability

Four overarching themes emerged: three themes broadly aligned with three of the four main constructs of the GTI framework (see Fig. 1) and the fourth described the wider policy context (see Table 5). Themes were schools’ capacity to sustain health interventions (GTI construct ‘capacity’), staff’s motivation and commitment (GTI construct ‘potential’), intervention adaptation and integration (GTI construct ‘capability’) and wider policy context for health promotion. We found that the fourth GTI construct of ‘contribution’ was implicated within the other themes (we highlight where this occurs) and comment on this further in the discussion. Themes and sub-themes are described below.

**Table 5:** Themes and sub-themes on the factors affecting the sustainability of health interventions in schools

Theme	Sub-themes	Sub-sub-themes	Reports that identified (sub)theme
Schools’ capacity to sustain health intervention—the social norms, roles and resources that affected whether schools could sustain an interventions	Educational outcomes took precedence over health promotion	N/A	[43, 44, 46, 48, 49, 52, 54, 56, 65]
	Staff roles in sustainability—how the professional roles of different staff contributed to sustainability processes.	The importance of the principal and school administration	[43, 45–48, 52–54, 59, 63–65]
		Teachers’ autonomy in the classroom	[43, 44, 48, 65]
	Funding and material resources—the availability of funding, materials and space for sustaining an intervention.	N/A	[45–49, 51, 52, 54–59, 63, 64, 66]
	Cognitive resources—schools’ access to staff with the knowledge and skills to continue to promote, co-ordinate and/or deliver the intervention.	Staff turnover—the need to train new staff and retain experienced and trained staff.	[43, 46–49, 51–53, 55, 56, 58, 59, 63–65]
		The importance of training	[43, 46–49, 51–53, 56, 59, 64, 65]
	Social resources—the resources that came from schools’ connections with other schools and organisations	N/A	[43, 45, 48, 58]
Staff motivation and commitment—factors influencing the intentions of staff to sustain an intervention	Observing and evaluating effectiveness	N/A	[43–50, 52, 55, 59, 63–66]
	Staff confidence in delivering health promotion	N/A	[43, 46–50, 63, 64]
	Parent support for the intervention	N/A	[43, 45, 46, 48, 52, 59, 62, 64, 65]
	Believing in the importance of the intervention	N/A	[43, 44, 46–49, 52, 63]
	The impact of school climate	N/A	[46, 54, 60, 63]
Intervention adaptation and integration—factors influencing whether it was operationally possible to sustain an intervention	The workability of the intervention—the work carried out to fit the intervention into existing school practices and routines.	Fitting the intervention into the time available	[44–49, 52–56, 58, 63–66]
		Matching the intervention to students’ needs	[43, 46, 53, 54, 63–65]
		The need for up-to-date materials	[48, 49, 53, 54, 64]
	The integration of the intervention into school policies and plans.	N/A	[43, 48, 63]
Wider policy context for health promotion—whether policies supported school health promotion	N/A	N/A	[43, 48, 52, 55–57, 62]

### Theme 1: Schools’ capacity to sustain health interventions

Schools’ social norms, staff roles, resources and systems were reported to influence sustainability. Five sub-themes developed from 20 studies of 14 interventions [43–49, 51–59, 63–66].

1. Educational outcomes took precedence over health promotion

Teachers, principals and administrators prioritised teaching the academic curriculum, meeting educational standards and regulations. Under time constraints, health promotion was considered dispensable, a theme that arose from nine studies (high and medium reliability) of six interventions focused on physical activity, healthy eating and mental health [43, 44, 46, 48, 49, 52, 56, 65]. A district-level informant from the CATCH study commented:

…if you’re going to prioritize, you’re going to prioritize on academics. ...You always concentrate on academics but there was more room for PE and health and those kinds of things before the state kicked in the really extremely rigorous academic standards. ([48], p. 515)

There were some exceptions where principals or administrators encouraged staff to focus on health [43, 46, 48], but the prevailing norm was to focus on academic attainment.

2. Staff members’ roles in sustainability

Staff members’ roles and autonomy were reported to affect whether interventions were sustained at school-level or solely by individual practitioners. Two deeper sub-themes emerged: the importance of the principal and administration, and teachers’ autonomy in the classroom.

i) *The importance of the principal and school administration*

Commitment and support from the principal and administration (including the school district in US studies) were considered crucial to ‘pave the way’ for sustainability [46], a sub-theme identified in 12 studies of 11 interventions [43, 45–48, 52–54, 59, 63–65]. Senior staff had the power to stop or continue an intervention at school-level through authorisation [46, 48], re-distributing school funds to or away from interventions [45, 47], allocating time for delivery [43, 46, 47] and providing training for new staff [43, 47, 63] (see sub-theme 4 (i) ‘Staff turnover’ in the ‘Theme 1: Schools’ capacity to sustain health interventions’ section).

Beyond resources, principals/administrators could demonstrate their commitment through integrating the intervention into school policies [43], recruiting new staff who were well-disposed to it [63], giving staff positive recognition [43, 53, 64] and managing staff to ensure that they continued [43]. The principal had a key role in continuing to enrol staff in a community of practice and persuading staff that it was right for them to address health [43]. This sub-theme overlaps with the GTI domain ‘cognitive participation’ under the construct ‘contribution’.

ii) *Teachers’ autonomy in the classroom*

Four studies of four interventions (high and medium reliability) indicated that teachers had autonomy to decide whether to sustain interventions in their classroom, within the bounds of the curriculum and principals’ leadership [43, 44, 48, 65]. Other studies revealed that if teachers sustained interventions, they could adapt them as they deemed appropriate (see sub-theme 1 ‘The workability of the intervention’ in the ‘Theme 3: Intervention adaptation and integration’ section). One teacher from a US study of CATCH reported [48]:

It is an individual decision. The state has a framework of what we are supposed to teach. We are asked to teach the things that the district recommends, but if you have more time, you can teach other things as well. No one has asked us to use the CATCH curriculum since the program ended in our school so it was up to us. ([48], p. 509)

There were some examples of collective action among teachers (reflecting GTI domain ‘collective action’ under ‘contribution’). Two US studies (medium and high reliability) of physical-activity interventions showed teachers working together to plan and develop ideas [44] and to encourage the principal to raise funds for sustainability [45]. There was an example of staff receiving logistical support [46] and providing internal training to other staff [48]. The piecemeal evidence for collective action may reflect the lack of attention given to this factor in the studies or a norm that teachers’ work with an intervention beyond the evaluation of effectiveness is typically independent.

3. Funding and material resources

Insufficient funding, equipment, materials and/or physical space could lead to discontinuation, cause logistical challenges [43, 47, 64] or become a reason for adaptation (see sub-theme 1 ‘The workability of the intervention’ in the ‘Theme 3: Intervention adaptation and integration’ section), a sub-theme developed from 16 studies of 11 interventions [45–49, 51, 52, 54–59, 63, 64, 66]. A lack of resources could motivate schools to seek out external funds via fundraising, grants or assistance from school-related associations [48, 57, 58, 66], redistribute school budgets [45] or find alternative means such as volunteers or parental payments [47, 57, 66]. As one study (medium reliability) of an all-girls physical-activity intervention reported:

Lack of finances was mentioned as a reason that teachers did not offer guest instructors or hold weekly lunch bunches. Whereas some teachers asked for volunteers to teach yoga or dance, others used videos or asked students to pay a $5 activities fee at the beginning of the class to use for guest instructors’ fees. ([47], p. 5)

4. Cognitive resources

Schools needed to retain the knowledge, skills and experience to sustain the intervention. Two deeper sub-themes emerged related to staff turnover and the importance of training.

i) *Staff turnover*

Fifteen studies of ten interventions described the adverse impact of staff turnover. As staff left, organisational knowledge, enthusiasm and the co-ordination of the intervention could dissipate [43, 46–49, 51–53, 55, 56, 58, 59, 63–65]. A change in principal [43, 48, 63] or loss of a champion (a senior staff member who advocated and assumed responsibility for intervention coordination and integrity) could jeopardise sustainability [46, 58, 62]. New decision-makers did not always share enthusiasm for the intervention or had other priorities, as a clinician from one highly reliable US study of a mental-health intervention explained:

We’ve lost a major senior administrator that is proactive and advocated for the kids’ needs, across the board, regular education and special education. Things have changed. Within the last year, they’re just looking at all the academics right now. ([46], p. 138)

ii) *The importance of training*

A lack of training for new teachers or booster training was a barrier to sustainability, a sub-theme emerging from 12 studies of nine interventions [43, 46–49, 51–53, 56, 59, 64, 65]. One Dutch study (medium reliability) of an intervention to reduce aggressive behaviour found a designated school co-ordinator to train and coach teachers facilitated sustainability [63]. As well as giving staff the skills and knowledge for delivery, training could generate enthusiasm and communicate the intervention’s philosophy [47, 48], as described by a teacher from a US study (medium reliability) of CATCH:

The staff development was interesting and motivated teachers. They learned about nutrition and fitness. They got excited about it and therefore implemented it. And that made it difficult to implement in schools that had not had the training. They missed a real motivational surge and missed looking at the importance and hearing from experts. ([48], p. 515)

5. Social resources

Schools’ networks with other schools, community organisations and funding agencies appeared to influence sustainability, a sub-theme emerging from four studies (high, medium and low reliability) of four interventions [43, 45, 48, 58]. Strong social links could give schools access to funding [58] and training [48], and collaborations with community organisations and other schools could motivate schools to maintain and develop interventions [43].

### Theme 2: Staff motivation and commitment

Five sub-themes emerged on staff motivation and commitment to sustain health interventions from 18 studies of 15 interventions [43–50, 52, 53, 55, 59, 60, 62–66].

1. Observing and evaluating effectiveness

Directly observing the benefits for students’ engagement, wellbeing and behaviour was a strong motivator to continue [43–50, 52, 63, 65, 66]. No staff referred to the findings of the effectiveness evaluation when discussing the intervention’s value, though a clinician in one study commented seeing a change in students based on a ‘pre and post test’ [46]. Conversely, negative responses from students could be a barrier [48, 55, 64]. For example, a teacher from a Dutch study (medium reliability) of an intervention to reduce aggressive behaviour reported:

It gives the team power. And, especially now, with more children with behavioral problems in the classroom. When you stay on the positive side, almost all children will get along. ([63], p. 85)

Two studies (high reliability) asked students about their experiences of physical activity interventions [45, 65] and found they had little decision-making power over what activities were sustained; they were willing participants, but opportunities were largely dictated by their families or the school. For example, a student commented on a component discontinued due to time constraints (as reported by teachers):

Taylor said, ‘We started these warmups, and then they stopped. I don’t know why, but I wish we had them. It is hard to run the CV day with no warmup.’ ([65], p. 114)

Only four studies (one high, two medium and one low reliability) of four interventions referred to more formal processes to appraise effectiveness [43, 46, 59, 63], overlapping with the GTI domain of ‘reflexive monitoring’ under ‘contribution’. Two studies found no differences in sustainability between schools with procedures for reviewing the intervention and those without [59, 63]. One study (medium reliability) reported principals who sustained the intervention regularly evaluated health-promotion activities.

2. Staff confidence in delivering health promotion

Staff who had been trained in the intervention felt more confident and better prepared to deliver it [47–49, 52, 64] (see sub-theme 4 (ii) ‘The importance of training’ in the ‘Theme 1: Schools’ capacity to sustain health interventions’ section). Teachers delivering an intervention outside of their usual expertise were less likely to sustain it [43, 47–50, 64, 65], for example, PE teachers delivering nutrition education [47] or classroom teachers delivering PE [43, 48–50, 65]:

Among classroom teachers, feeling inadequately prepared to implement PE was frequently reported; and in many cases, teachers had little interest in gaining the skill. ([49], p. 471)

3. Parent support

Five studies noted parent support in a general sense was helpful [43, 45, 52, 59, 64]. Four studies covered parent support in more depth; staff indicated how lack of parent support could reduce their motivation to sustain an intervention [46, 48, 62, 65]. This sub-theme overlaps with the GTI domain ‘coherence’ under ‘contribution’. A teacher from an English study (high reliability) of a physical-activity intervention explained:

I think a lot of it is home life, if the parents don’t push them towards sporting activities then you’re fighting a battle straight away in school. ([65], p. 8)

4. Believing in the importance of the intervention

Belief in the importance of the intervention motivated staff to sustain it, a sub-theme arising from seven studies of six interventions [43, 44, 46–49, 52, 63] and was related to the importance of training (sub-theme 4 (ii) ‘The importance of training’ in the ‘Theme 1: Schools’ capacity to sustain health interventions’ section) and observing intervention effectiveness (sub-theme 1 ‘Observing and evaluating effectiveness’ in the ‘Theme 2: Staff motivation and commitment’ section). Principals who reported sustaining a 3-year HPS intervention in Norway, which aimed to create a positive school environment for health, were keen to communicate its importance:

School satisfaction and safety are at the bottom of this school. It is under the teachers’ skin and in our walls. We work with this no matter what is on our agenda. ([43], p. 59)

5. The impact of school climate

There was limited evidence on the impact of staff perception of the school climate. One highly reliable US study of CATCH suggested climate might differentially impact on different interventions: a positive climate was associated with more teaching hours of the CATCH curriculum but higher levels of saturated fat in school meals [60]. Respondents in two other studies (medium and high reliability) reported that a negative climate meant that sustainability processes were superseded by more critical organisational priorities [46, 63]. One US study (low reliability) of a workplace health-and-safety intervention found no relationship between climate and sustainability.

### Theme 3: Intervention adaptation and integration

Schools’ ability to sustain an intervention was affected by its ‘workability’—the degree to which it could be shaped into existing school practices and routines, and its integration into school policies and plans. These two sub-themes emerged from 18 studies of 13 interventions [43–49, 52–56, 58, 60, 63–66].

1. The workability of the intervention

Three deeper sub-themes transpired: fitting the intervention into the time available, matching the intervention to students’ needs and the need for up-to-date equipment and materials.

i) *Fitting the intervention into the time available*

Frequently, staff identified that interventions required too much time, time which was primarily devoted to delivering the curriculum (see sub-theme 1 ‘Educational outcomes took precedence over health promotion’ in the ‘Theme 1: Schools’ capacity to sustain health interventions’ section) [44–46, 48, 49, 52–56, 63–65]. Staff dealt with time constraints by reducing or dropping components [45, 47, 64, 65], or making time for the intervention by adapting it to classroom routines [44, 50] or incorporating elements of it into the existing curriculum [48, 52, 53, 56, 58, 65].

ii) *Matching the intervention to students’ needs*

Adaptation was also important to match the needs of different cohorts of students, to offer the intervention to different grades [53, 63], better fit students’ learning abilities or make lessons more contextually relevant [43, 54], devote more time to particular activities to ensure students understood a subject or better engage students [46, 64].

iii) *The need for up-to-date materials*

Over time, new equipment and materials were needed as equipment grew worn or was lost [49], materials became dated [48, 53, 64], new technological advances emerged [50, 64] or adaptations were needed to meet students’ needs [53, 54, 64].

2. Integration of the intervention with school policies and plans

One Dutch study of an intervention to reduce aggressive behaviour and one Norwegian study of an HPS intervention (medium reliability) reported that schools with greater sustainability more often made reference to it in school policies or plans [43, 63]. Studies suggested formal documentation signalled principals’ and administrators’ commitment to the intervention [63], legitimised it [48, 63], made staff accountable [43] or made the intervention resilient to staff turnover [43] (see sub-theme 4 (i) ‘Staff turnover’ in the ‘Theme 1: Schools’ capacity to sustain health interventions’).

### Theme 4: Wider policy context for health promotion

The wider policy context could also affect sustainability, a thematic area positioned outside of the GTI framework, emerging from seven studies of five interventions. Regional or national health policies could support sustainability by legitimising health promotion in schools’ policies [43, 48] (see sub-theme 1 ‘Educational outcomes took precedence over health promotion’ in the ‘Theme 1: Schools’ capacity to sustain health interventions’ section). Over time, health policies could shape social norms: for example, increasing tobacco-control regulations could enhance the sustainability of outdoor-smoking bans in schools [62]. Policy could also provide funding and resources [55, 57], though additional resources could also lead to competing interventions, potentially displacing existing ones [55, 56].

## Discussion

### Summary of key findings

The sustainability of public health interventions after start-up funding and/or other resources end has been relatively uncharted in schools compared to health care. We identified 24 studies assessing the sustainability of school-based health interventions delivered partly/wholly by educators or school-employed health professionals, but quality was not consistently high. None of the interventions assessed were fully sustained; all had components sustained by some schools or staff, bar one that was completely discontinued. Identifying common facilitators and barriers could help researchers and providers optimise the sustainability of school interventions, and consider whether/how the intervention is likely to have a lasting impact on student and staff health. Two key facilitators emerged. First is the central importance of a committed principal and administration that could authorise continuation, allocate resources, integrate the intervention into school policies and enrol new staff into a community of practice. Second is the importance of supporting staff who are confident in delivering health promotion and believe in its value. These facilitators are consistent with studies of the implementation of school health interventions [13, 31, 67], suggesting factors are crucial to both phases.

Many of the facilitators and barriers to sustainability identified for school settings were similar to those in health care: for example, dedicated leaders, the need for continued resources and training, staff turnover and intervention workability [21, 24–27]. Several factors were more salient for schools. Health encompasses multiple outcomes, some of which may be more obviously relevant to school settings. We identified the sub-theme of educational outcomes taking precedence over physical activity, nutrition and mental health interventions, but not for those focused on anti-social or violent behaviour. This suggests that throughout adoption and implementation, change agents need to convince schools that health interventions can bring education benefits [30, 68–70].

Student engagement was key to implementation and sustainability at teacher-level. A central role of educators is to engage students [29, 71], and staff were unlikely to sustain interventions that did not draw students in [48]. Sometimes sustainability was prompted by students’ requests for the intervention [44, 45]. Knowing parents encouraged the healthy activities of the intervention outside of school also motivated staff to continue, further supporting the view that schools are complex adaptive systems, where multiple networks of agents act and react to one another [30]. In contrast, only 16% of the 62 sustainability approaches in Lennox et al.’s review [23] included patient involvement, suggesting that most existing tools and frameworks for health care settings do not consider patient support for the intervention critical for sustainability.

Also of particular significance for schools was the need to adapt intervention materials and activities to accommodate other curriculum requirements and the diversity of children’s backgrounds and development [29, 72]. This dynamic context suggests that intervention developers should anticipate the need for adaptation, even for effective, well-implemented and funded school health interventions [21, 30, 73].

Contrary to other studies of sustainability in health care settings [20], we found little evidence that champions helped sustain interventions: like other staff, champions moved to new institutions leaving interventions at risk. We found no discernible relationship between evidence of effectiveness and sustainability, and no school staff mentioned outcome evaluation as an influential factor in sustainability.

### Strengths and limitations

Our review was comprehensive and rigorously conducted. It is the first to apply the GTI to the study of sustainability. We found the framework helpful in creating a balance between listing the common enablers and barriers and representing the complexity and context-dependent nature of sustainability in schools. The data aligned well with the constructs of capacity (theme 1), potential (theme 2) and capability (theme 3), while the construct of contribution was implicated within the other themes. It made sense to consider ‘cognitive participation’ and ‘collective action’ under the construct of ‘capacity’ as the ongoing enrolment of staff, the legitimisation of health activities, and whether staff worked independent or collectively appeared significantly affected by schools’ social norms and roles. Under capacity, we included an additional domain of ‘social resources’ which suggested that contact between schools and other organisations could facilitate sustainability through creating opportunities for resource- and knowledge-sharing, while stimulating ongoing interest in the intervention.

Regarding limitations, we did not double-screen full reports and we may have missed reports due to the array of terms used to describe sustainability, despite our sensitive search strategy. We deviated from our original protocol in using thematic synthesis rather than meta-ethnography due to the nature of studies found. We excluded interventions delivered by clinical services co-located in schools, and consequently, our findings may be less representative of the sustainability of targeted or tiered services which typically require a high level of clinical expertise (only 3 of the 24 interventions in the review were targeted). The sustainability of health interventions provided solely by external clinicians is unknown; for example, they could be more sustainable because they do not require educators to expend time gaining additional knowledge and skills, or they may be less because they require sustained funding. There was substantial heterogeneity in study designs, methods and reporting of included studies; many studies were methodologically weak and did not report on the sustainability of all components, in particular reporting for family/community components was poor. Most studies were located in the US, and consequently, our review findings may be most relevant to this setting. Around half of interventions focused on healthy eating/physical activity, with a lack of evidence for the sustainability of other public-health interventions.

### Implications for research and policy

Informed by our synthesis, we propose three questions to consider when optimising school health interventions. First, is it important that each component is sustained? Some components, such as needs assessment, may be time-limited stepping-stones. Second (if a component is to be sustained), how would you expect the intervention to be sustained: if there were high staff turnover or the loss of the champion, during time-pressured periods such as exams, with different classes of students with varying needs or if there were no opportunities for regular training updates? Third, do staff understand the key theoretical principles that should underpin any adaptations to intervention activities and resources? Creating forums during the period of the evaluation of effectiveness when these ‘stress-testing’ questions can be discussed with staff could help researchers to understand the likely sustainability of interventions.

Stronger study designs/methodology are needed for future research; there were few longitudinal studies prospectively following intervention sustainability from initial implementation. Increased use of conceptual theory would enhance studies’ richness and breadth and improve the analytic generalisability of findings. Student engagement in the intervention should be considered a key factor affecting both implementation and sustainability processes. The inclusion of views from a range of school participants, including students, would strengthen the validity of findings. Improved reporting on sustainability of *all* intervention components is key, with justification provided for excluding specific components. Research on the sustainability of interventions outside health eating/physical activity is needed, for example, there were no studies of sexual-health interventions, as are studies of the sustainability of interventions delivered by external providers co-located in schools.

Sustainability strategies contributed to our analysis where authors commented on them in papers’ results and discussions [43–45, 52, 64]. However, several papers referred to specific sustainability strategies in their background sections but did not consider their impact in their analysis of sustainability, including ‘train-the-trainer’ models to spread the intervention across and between schools [58, 63], external consultants exploring adaptations with staff [53] and a staged-approach to implementation [50]. Primary research on the impact of implementation and sustainability strategies and planning would be valuable [74, 84].

Our review suggests regional and/or national school policies and educational standards that promote health and wellbeing and its connection to students’ learning and school enjoyment could enhance sustainability by legitimising staff spending time, effort and resources on continuation, as well as bringing funding and resources to sustain health goals.

## Conclusion

Multiple factors facilitating and prohibiting schools’ ability to sustain health interventions emerged from the review, and existing evidence suggests sustainability depends upon schools developing and retaining senior leaders and staff that are knowledgeable, skilled and motivated to continue delivering health promotion through ever-changing circumstances. Evidence of intervention effectiveness did not appear to be an influential factor. However, there is a significant gap in our understanding of how to sustain interventions and methodologically stronger primary research, informed by theory, is needed.

## Supplementary information

**Additional file 1:** Search terms for each database.

**Additional file 2:** Website search results.

**Additional file 3:** Contact with subject experts.

**Additional file 4:** Data extraction and quality appraisal form.

**Additional file 5:** Quality appraisal guidance and ratings.

**Additional file 6:** PRISMA reporting standards.

**Additional file 7:** Additional details on sustainability study design participants.

## Data Availability

The data extraction forms used and analysed during the current study are available from the corresponding author on reasonable request.

## Abbreviations

ASAP: Adolescent Suicide Awareness Program
BMI: Body mass index
CATCH: Child and Adolescent Trial for Cardiovascular Health
CBITS: Cognitive Behavioral Intervention for Trauma in Schools
F&V: Fruit and vegetables
GBG: Good Behavior Game
GTI: General Theory of Implementation
HOPE: Health Optimizing PE
HPS: Health Promoting Schools
MVPA: Moderate-to-Vigorous Physical Activity
OECD: Organisation for Economic Co-operation and Development
PA: Physical activity
PDV: Physical dating violence
PE: Physical education
PTSD: Post-traumatic stress disorder
WHO: World Health Organization
