# Supplementary material for: The sustainability of public health interventions in schools: a systematic review
Source: Implement Sci. 2020 Jan 6;15:4. doi: 10.1186/s13012-019-0961-8 (PMC6945701; doi:10.1186/s13012-019-0961-8)
Supplement: Supplementary file 1 — Additional file 1: Search terms for each database. [file 13012_2019_961_MOESM1_ESM.docx]

**Additional file 1: Search terms for each database**

**ERIC and British Education Index**

There were no thesaurus terms for sustainability. Searches were conducted in the BEI and ERIC on 5^th^ September 2017 and combined the following terms for sustainability, school, intervention and public health:

TI(sustain* OR continua* OR maintenance OR institutionalisation OR institutionalization OR routinisation OR routinization OR embed* OR incorporation OR integration OR normalization OR stabilization OR durability OR “long-term implementation” OR “long term implementation” OR discontinuation OR mainstreaming OR scale-up OR “scale up” OR scaling-up OR “scaling up” OR endurance OR persistence) OR AB(sustain* OR continua* OR maintenance OR institutionalisation OR institutionalization OR routinisation OR routinization OR embed* OR incorporation OR integration OR normalization OR stabilization OR durability OR “long-term implementation” OR “long term implementation” OR discontinuation OR mainstreaming OR scale-up OR “scale up” OR scaling-up OR “scaling up” OR endurance OR persistence)

AND

SU(Schools) OR TI (school* OR student* OR pupil* OR teacher* OR “teaching staff” OR “teaching personnel” OR school-based OR “school based”) OR AB (school* OR student* OR pupil* OR teacher* OR “teaching staff” OR “teaching personnel” OR school-based OR “school based”)

AND

SU(“EDUCATIONAL evaluation” OR “PROGRAM development (Education)” OR “PROGRAM implementation (Education)” OR “PROGRAM improvement (Education)” OR “PROGRAM effectiveness (Education)” OR “PROGRAM design (Education)” OR PROGRAM attitudes (Education)” OR “ORGANIZATIONAL change – Study & teaching”)

OR TI (intervention* OR program* OR “organizational change” OR “organisational change” OR “change process*” OR “organizational transformation” OR “organisational transformation” OR innovation*) OR AB (intervention* OR program* OR “organizational change” OR “organisational change” OR “change process*” OR “organizational transformation” OR “organisational transformation” OR innovation*)

AND

SU(“HEALTH promotion” OR “CHILDREN – Health” OR “STUDENTS – Health” OR “SCHOOL children – Health” OR “SCHOOL health services” OR “HEALTH education” OR “HEALTH programs” OR “LIFE skills”) OR TI (“health policy” OR “health policies” OR “health environment*” OR “healthy environment*” OR “health ethos” OR “health attitude*” OR “healthy attitude*” OR “health curricul*” OR “health behav*” OR “healthy behav*” OR “health intervention*” OR “physical activity” OR “sedentary behav*” OR eating OR tobacco OR alcohol OR “substance abuse” OR bullying OR aggressi* OR safety OR violence OR “mental health” OR wellbeing OR “sexual health” OR “sex education”) OR AB (“health policy” OR “health policies” OR “health environment*” OR “healthy environment*” OR “health ethos” OR “health attitude*” OR “healthy attitude*” OR “health curricul*” OR “health behav*” OR “healthy behav*” OR “health intervention*” OR “physical activity” OR “sedentary behav*” OR eating OR tobacco OR alcohol OR “substance abuse” OR bullying OR aggressi* OR safety OR violence OR “mental health” OR wellbeing OR “sexual health” OR “sex education”)

**ERIC records identified n=1,268**

**BEI records identified n=71**

**CINAHL**

There was no suitable Major or Minor Subject Heading (MH) in CINAHL for sustainability. The search was conducted on the 22^nd^ September 2017 and combined the following terms for sustainability, school, intervention and public health:

(TI (sustain* OR continua* OR maintenance OR institutionalisation OR institutionalization OR routinisation OR routinization OR embed* OR incorporation OR integration OR normalization OR stabilization OR durability OR “long-term implementation” OR “long term implementation” OR discontinuation OR mainstreaming OR scale-up OR “scale up” OR scaling-up OR “scaling up” OR endurance OR persistence)) OR (AB (sustain* OR continua* OR maintenance OR institutionalisation OR institutionalization OR routinisation OR routinization OR embed* OR incorporation OR integration OR normalization OR stabilization OR durability OR “long-term implementation” OR “long term implementation” OR discontinuation OR mainstreaming OR scale-up OR “scale up” OR scaling-up OR “scaling up” OR endurance OR persistence))

AND

(MH “Students, High School”) OR (MH “Students, Middle School”) OR (MH “Schools, Elementary”) OR (MH “Schools, Middle”) OR (MH “Schools, Secondary”) OR (MH “Teachers”) OR (TI (school* OR student* OR pupil* OR teacher* OR “teaching staff” OR “teaching personnel” OR school-based OR “school based”)) OR (AB (school* OR student* OR pupil* OR teacher* OR “teaching staff” OR “teaching personnel” OR school-based OR “school based”))

AND

(MH “Intervention Trials”) OR (MH “Program Development) OR (MH “Program Evaluation”) OR (MH “Organizational Change”) OR (TI (intervention* OR program* OR “organizational change” OR “organisational change” OR “change process*” OR “organizational transformation” OR “organisational transformation” OR innovation*)) OR (AB (intervention* OR program* OR “organizational change” OR “organisational change” OR “change process*” OR “organizational transformation” OR “organisational transformation” OR innovation*))

AND

(MH “Public Health”) OR (MH “Accidents+”) OR (MH “Exposure to Violence”) OR (MH “Hygiene”) OR (MH “Public Health Dentistry+”) OR (MH “Safety+”) OR (MH “Reproductive Health”) OR (MH “School Health”) OR (MH “Social Determinants of Health”) OR (MH “Women’s Health”) OR (MH “Health Promotion”) OR (MH “Health Education+”) OR (MH “Habits+”) OR (MH “Eating Behavior+”) OR (MH “Drinking Behavior+”) OR (MH “Attitude to Health) OR (MH “Health Beliefs”) OR (MH “Life Style Changes”) OR (MH “Life Style, Sedentary”) OR (TI (“health policy” OR “health policies” OR “health environment*” OR “healthy environment*” OR “health ethos” OR “health attitude*” OR “healthy attitude*” OR “health curricul*” OR “health behav*” OR “healthy behav*” OR “health intervention*” OR “physical activity” OR “sedentary behav*” OR eating OR tobacco OR alcohol OR “substance abuse” OR bullying OR aggressi* OR safety OR violence OR “mental health” OR wellbeing OR “sexual health” OR “sex education”)) OR (AB (“health policy” OR “health policies” OR “health environment*” OR “healthy environment*” OR “health ethos” OR “health attitude*” OR “healthy attitude*” OR “health curricul*” OR “health behav*” OR “healthy behav*” OR “health intervention*” OR “physical activity” OR “sedentary behav*” OR eating OR tobacco OR alcohol OR “substance abuse” OR bullying OR aggressi* OR safety OR violence OR “mental health” OR wellbeing OR “sexual health” OR “sex education”))

**CINAHL records identified n=46**

**EMBASE**

The search was conducted in EMBASE on the 15^th^ September 2017 and combined the following terms for sustainability, school, intervention and public health:

(program sustainability).sh. OR sustain*.ti,ab. OR continua*.ti,ab. OR maintenance.ti,ab. OR institutionalisation.ti,ab. OR institutionalization.ti,ab. OR routinisation.ti,ab. OR routinization.ti,ab. OR embed*.ti,ab. OR incorporation.ti,ab. OR integration.ti,ab. OR normalization.ti,ab. OR stabilization.ti,ab. OR durability.ti,ab. OR (long-term implementation).ti,ab. OR (long term implementation).ti,ab. OR discontinuation.ti,ab. OR mainstreaming.ti,ab. OR scale-up.ti,ab. OR (scale up).ti,ab. OR scaling-up.ti,ab. OR (scaling up).ti,ab. OR endurance.ti,ab. OR persistence

AND

school.sh. OR (high school).sh. OR kindergarten.sh. OR (middle school).sh. OR (primary school).sh. OR school*.ti,ab. OR student.sh. OR (elementary student).sh. OR (high school student).sh. OR (middle school student).sh. OR pupil*.ti,ab. OR teacher.sh. OR (school teacher).sh. OR teacher*.ti,ab. OR school-based.ti,ab. OR (school based).ti,ab.

AND

(intervention study).sh. OR (program development).sh. OR exp program evaluation/ OR health program.sh. OR intervention*.ti,ab. OR program*.ti,ab. OR (organizational change).ti,ab. OR (organisational change).ti,ab. OR (change process*).ti,ab. OR (organizational transformation).ti,ab. OR (organisational transformation).ti,ab. OR innovation*.ti,ab.

AND

(public health).sh. OR (health promotion).sh. OR (health education).sh. OR (school health education).sh. OR exp health behavior/ OR prevention.sh. OR accident prevention.sh. OR wellbeing.sh. OR (physical well-being).sh. OR (psychological well-being).sh. OR (health adj3 (promot* or policy or policies or educat* or environment* or ethos* or attitude* or curricul* or behav* or intervention*)).ti,ab. OR (healthy environment*).ti,ab. OR (healthy attitude*).ti,ab. OR (healthy behav*).ti,ab. OR (physical activity).ti,ab. OR (sedentary behav*).ti,ab. OR eating.ti,ab. OR tobacco.ti,ab. OR alcohol.ti,ab. OR (substance abuse).ti,ab. OR bullying.ti,ab. OR aggressi*.ti,ab. OR safety.ti,ab. OR violence.sh. OR exp mental health/ OR wellbeing.ti,ab. OR (sexual health).ti,ab. OR (sexual education).sh.

**EMBASE records identified n=4,085**

**PSYCINFO**

The search was conducted in EMBASE on the 12^th^ September 2017 and combined the following terms for sustainability, school, intervention and public health:

sustain*.ti,ab. OR continua*.ti,ab. OR maintenance.ti,ab. OR institutionalisation.ti,ab. OR institutionalization.ti,ab. OR routinisation.ti,ab. OR routinization.ti,ab. OR embed*.ti,ab. OR incorporation.ti,ab. OR integration.ti,ab. OR normalization.ti,ab. OR stabilization.ti,ab. OR durability.ti,ab. OR (long-term implementation).ti,ab. OR (long term implementation).ti,ab. OR discontinuation.ti,ab. OR mainstreaming.ti,ab. OR scale-up.ti,ab. OR (scale up).ti,ab. OR scaling-up.ti,ab. OR (scaling up).ti,ab. OR endurance.ti,ab. OR persistence

AND

exp schools/ OR school*.ti,ab. OR (School Environment).sh. OR Students.sh. OR exp Elementary School Students/ OR (High School Students).sh. OR (Kindergarten Students).sh. OR (Junior High School Students).sh. OR pupil*.ti,ab. OR exp Educational Personnel/ OR (Elementary School Teachers).sh. OR (High School Teachers).sh. OR (Junior High School Teachers).sh. OR (Middle School Teachers).sh. OR teacher*.ti,ab. OR school-based.ti,ab. OR (school based).ti,ab.

AND

intervention.sh. OR (school based intervention).sh. OR exp Program Development/ OR exp Program Evaluation/ OR (organizational change).sh. OR intervention*.ti,ab. OR program*.ti,ab. OR (organizational change).ti,ab. OR (organisational change).ti,ab. OR (change process*).ti,ab. OR (organizational transformation).ti,ab. OR (organisational transformation).ti,ab. OR innovation*.ti,ab.

AND

(Public Health).sh. OR (Community Health).sh. OR exp Health Promotion/ OR exp Health Behavior/ OR exp Health Education/ OR exp Prevention/ OR (Well Being).sh. OR exp Drug Usage Attitudes/ OR (health adj3 (promot* or policy or policies or educat* or environment* or ethos* or attitude* or curricul* or behav* or intervention*)).ti,ab. OR (healthy environment*).ti,ab. OR (healthy attitude*).ti,ab. OR (healthy behav*).ti,ab. OR (physical activity).ti,ab. OR (sedentary behav*).ti,ab. OR eating.ti,ab. OR tobacco.ti,ab. OR alcohol.ti,ab. OR (substance abuse).ti,ab. OR bullying.ti,ab. OR aggressi*.ti,ab. OR safety.ti,ab. OR violence.sh. OR (Mental Health).sh. OR wellbeing.ti,ab. OR (sexual health).ti,ab. OR (Sex Education).sh.

**Psychinfo records identified n=2,107**

**PUBMED**

There was no suitable Medical Subject Heading (MH) in PubMed for sustainability. The search was conducted on the 19^th^ September 2017 and combined the following terms for sustainability, school, intervention and public health:

sustain*[TIAB] OR continua*[TIAB] OR maintenance[TIAB] OR institutionalisation[TIAB] OR institutionalization[TIAB] OR routinisation[TIAB] OR routinization[TIAB] OR embed*[TIAB] OR incorporation[TIAB] OR integration[TIAB] OR normalization[TIAB] OR stabilization[TIAB] OR durability[TIAB] OR “long-term implementation”[TIAB] OR “long term implementation”[TIAB] OR discontinuation[TIAB] OR mainstreaming[TIAB] OR scale-up[TIAB] OR “scale up”[TIAB] OR scaling-up[TIAB] OR “scaling up”[TIAB] OR endurance[TIAB] OR persistence[TIAB]

AND

“School Health Services”[MH] OR “School Teachers”[MH] OR school*[TIAB] OR student*[TIAB] OR pupil*[TIAB] OR teacher*[TIAB] OR “teaching staff”[TIAB] OR “teaching personnel”[TIAB] OR school-based[TIAB] OR “school based”[TIAB]

AND

“Clinical Trials, Phase IV as Topic”[MH] OR “Program Evaluation”[MH] OR “Program Development”[MH] OR “Organizational Innovation”[MH] OR intervention*[TIAB] OR program*[TIAB] OR “organizational change”[TIAB] OR “organisational change”[TIAB] OR “change process*”[TIAB] OR “organizational transformation”[TIAB] OR “organisational transformation”[TIAB] OR innovation*[TIAB]

AND

“Health Promotion”[MH] OR “Health Education”[MH] OR “Health Knowledge, Attitudes, Practice”[MH] OR “Healthy Lifestyle”[MH] OR “health policy”[TIAB] OR “health policies” [TIAB] OR “health environment*”[TIAB] OR “healthy environment*”[TIAB] OR “health ethos”[TIAB] OR “health attitude*”[TIAB] OR “healthy attitude*”[TIAB] OR “health curriculum”[TIAB] OR “health curricula”[TIAB] OR “health behaviour*”[TIAB] OR “health behaviour*”[TIAB] OR “healthy behavior*”[TIAB] OR “healthy behaviour*”[TIAB] OR “health intervention*” [TIAB] OR “physical activity”[TIAB] OR “sedentary behavior*”[TIAB] OR “sedentary behaviour*”[TIAB] OR eating[TIAB] OR tobacco[TIAB] OR alcohol[TIAB] OR “substance abuse”[TIAB] OR bullying[TIAB] OR aggression[TIAB] OR aggressive[TIAB] OR safety[TIAB] OR violence[TIAB] OR “mental health”[TIAB] OR wellbeing[TIAB] OR “sexual health”[TIAB] OR “sex education”[TIAB]

**PubMed records identified n=2,780**

**WEB OF SCIENCE Social Sciences Citation Index and Conference Proceedings Citation Index – Social Science & Humanities (CPCI-SSH)**

Web of Science does not have a thesaurus. Topic Searches (TS) look for topic terms in the title, abstract and keywords. The search was conducted on the 19^th^ September 2017 and combined the following terms for sustainability, school, intervention and public health:

TS=(sustain* OR continua* OR maintenance OR institutionalisation OR institutionalization OR routinisation OR routinization OR embed* OR incorporation OR integration OR normalization OR stabilization OR durability OR “long-term implementation” OR “long term implementation” OR discontinuation OR mainstreaming OR scale-up OR “scale up” OR scaling-up OR “scaling up” OR endurance OR persistence)

AND

TS=(school* OR students OR pupil* OR “educational personnel” OR teacher* OR “teaching staff” OR “teaching personnel” OR school-based OR “school based”)

AND

TS=(intervention* OR program* OR “organizational change” OR “organisational change” OR “change process*” OR “organizational transformation” OR “organisational transformation” OR innovation*)

AND

TS=(“public health” OR prevention OR “health knowledge” OR “lifestyle changes” OR “health promot*” OR “health policy” OR “health policies” OR “health educat*” OR “health environment*” OR “healthy environment” OR “health ethos*” OR “health attitude*” OR “healthy attitude*” OR “health curricul*” OR “health behav*” OR “healthy behav*” OR “health intervention*” OR “physical activity” OR “sedentary behav*” OR eating OR tobacco OR alcohol OR “substance abuse” OR bullying OR aggressi* OR safety OR violence OR “mental health” OR wellbeing OR “sexual health” OR “sex education”)

**Web of Science records identified n=3,624**
