# Supplementary material for: The sustainability of public health interventions in schools: a systematic review
Source: Implement Sci. 2020 Jan 6;15:4. doi: 10.1186/s13012-019-0961-8 (PMC6945701; doi:10.1186/s13012-019-0961-8)
Supplement: Supplementary file 2 — Additional file 2: Website search results. [file 13012_2019_961_MOESM2_ESM.docx]

**Additional file 2: Website search results**

Standard search terms used when websites provided a search engine

sustain* OR continua* OR maintenance OR institutionalisation OR institutionalization OR routinisation OR routinization OR embed* OR incorporation OR integration OR normalization OR stabilization OR durability OR “long-term implementation” OR “long term implementation” OR discontinuation OR mainstreaming OR scale-up OR “scale up” OR scaling-up OR “scaling up” OR endurance OR persistence

Health-related search terms used in Eppi-Centre database

public health OR health promotion OR health education OR health behavior OR prevention OR wellbeing OR health policy OR health policies OR school environment OR school ethos OR health attitude* OR health curriculum OR health intervention OR healthy environment OR healthy attitude OR healthy behavior OR physical activity OR sedentary behav* OR eating OR tobacco OR alcohol OR substance abuse OR bullying OR aggressi* OR safety OR violence OR mental health OR sexual health OR sexual education

| **Website** | **Date searched** | **Comments** | **Studies identified?** |
| --- | --- | --- | --- |
| Australian Health Promoting Schools Association (https://www.achper.org.au/) | 27/06/18 | Searched contents of Curriculum Studies in Health and Physical Education journal. Screened title and abstract (T&A) of two most recent issues of Active + Healthy Journal – not possible to view contents pages of older issues as a non-member. Put standard search terms into website search. | None. |
| Barnardo’s (http://www.barnardos.org.uk/  what_we_do/policy_research_unit) | 27/06/18 | Searched policy and research documents. | None. |
| Center for Disease Control (www.cdc.gov) | 27/06/18 | Looked at the Adolescent and School Health are of website ([www.cdc.gov/healthyyouth/data/index.htm](http://www.cdc.gov/healthyyouth/data/index.htm)), including SHPPS and YRBSS survey results, and journal articles (2013-18). | None. |
| Education Endowment Foundation (https://educationendowmentfoundation.org.uk) | 29/06/18 | Looked at completed projects and scaling-up area of the website. | None. |
| EPPI-Centre database of education research  (https://eppi.ioe.ac.uk/webdatabases/Intro.aspx?ID=6) | 29/06/18 | Searched with the standard search terms using the free-text search. Identified 203 studies. Combined with health-related terms – 18 studies identified. Screened on title and then abstract if title looked promising. | None. |
| Institute for Effective Education (https://the-iee.org.uk/) | 29/06/18 | Searched through the “what we do” categories and each category’s linked website. Put the standard search terms into the website’s search engine. | None. |
| International School Health Network (www.internationalschoolhealth.org) | 2^nd^ and 3/07/19 | Searched the ‘Schools for all’ knowledge exchange program (<http://www.schools-for-all.org/>) using the term “sustain*”only as it was not possible to combine search terms (i.e. use the standard search terms) and there were a high number of links to search through. 134 links identified, looked at the first 100 links for references for research papers on sustainability.  Through this page [Home - where evidence meets experience](http://www.schools-for-all.org/page/Home+-+where+evidence+meets+experience) > [Glossary of Terms (GT)](http://www.schools-for-all.org/page/Glossary+of+Terms+%28GT%29) > [Implement, Maintain, Scale Up, Sustain & Capacity](http://www.schools-for-all.org/page/Implement%2C+Maintain%2C+Scale+Up%2C+Sustain+%26+Capacity) > [Sustainability and Sustainable Programs (GT)](http://www.schools-for-all.org/page/Sustainability+and+Sustainable+Programs+%28GT%29) - <http://www.schools-for-all.org/page/Sustainability+and+Sustainable+Programs+%28GT%29> – identified the following:  Han, S.S., Weiss, B. (2005). Sustainability of teacher implementation of school-based mental health programs. *J Abnorm Child Psychol, 33(6)*, 665-679. EXCLUDED ON DUPLICATION - INDENTIFIED IN THE DATABASE SEARCH  Harvey, G. (2005). *An Examination of the Sustainability of School Based Program Initiatives: The Case of “Turning the Tide in Schools*. University of Melbourne. Unpublished Doctoral Thesis. INCLUDED ON TITLE AND ABSTRACT. EMAILED AUTHOR TO ASK FOR A COPY ON 3^RD^ JULY. HE REPLIED THE SAME DAY AND GAVE ME A COPY. EXCLUDED AS SCHOOLS STILL RECEIVE SOME (REDUCED) FUNDING AND ASSISTANCE WITH INTERVENTION SO CONSIDERED A STUDY OF LONG-TERM IMPLEMENTATION.   O’Loughlin, J., Renauld, L., Richalrd, L., Gomez, L.S., Paradis, G. (1998). Correlates of the sustainability of community-based heart health promotion interventions. *Prev Med, 27(5 Pt 1),* 702-12. EXCLUDED ON DUPLICATION - IDENTIFIED IN THE DATABASE SEARCH.  Pluye, P., Potvin, L., Denis, J. L., Pelletier, J. (2004). Program sustainability: Focus on organizational routines.*Health Promotion International, Dec;19*(4), 489-500. EXCLUDED ON INTERVENTION – NOT SCHOOL-BASED.  Rissel, C., Finnegan, J., Bracht, N. (1995). Evaluating quality and sustainability: Issues and insights from the Minnesota Heart Health Program. *Health Promotion International,* 10, 199-207 EXCLUDED ON DATE.  Sanders, K.E., Francis, K., Lum, M., Schiada, G. (2004). Toward a grounded theory of sustainability in social service organizations: A systems point of view. *Systems Research and Behavioral Science, 27(5)*, 567-578 EXCLUDED ON INTERVENTION – NOT SCHOOL-BASED.  Scheirer, M. (2005). Is sustainability possible? A review and commentary on empirical studies of program sustainability. *American Journal of Evaluation, 26(3)*, 320-347. EXCLUDED ON EVIDENCE – NOT EMPIRICAL.  St. Leger, L. (2005). Questioning sustainability in health promotion projects and programs.*Health Promotion International, Dec;20*(4), 317-319. EXCLUDED ON EVIDENCE – NOT EMPIRICAL.  Swerissen, H., Crisp, B.R. (2004). The sustainability of health promotion interventions for different levels of social organizations. Health Promotion International, 19(1), 123-130. EXCLUDED ON EVIDENCE – NOT EMPIRICAL.  Through this page -Home - where evidence meets experience > [ISHN Conferences & Symposia](http://www.schools-for-all.org/page/ISHN+Conferences+%26+Symposia) > Integrating Health & Social Programs Within Education Systems: A Global Dialogue/European Discussion > [Forum Documents & Report](http://www.schools-for-all.org/page/Forum+Documents+%26+Report) <http://www.schools-for-all.org/page/Forum+Documents+%26+Report> - identified:   - Simovska, V., & McNamarra, P. (Eds.) (2015). *Schools for health and sustainability: theory, research and practice*. Dodrecht: Springer Science+Business Media B.V.[10.1007/978-94-017-9171-7](http://dx.doi.org/10.1007/978-94-017-9171-7)   And then looked at the chapters and screened the following on abstract:   - Mannix-McNamara , P., & Simovska, V. Schools for health and sustainability: insights from the past, present and for the future. p3-17   EXCLUDED ON EVIDENCE – NOT EMPIRICAL. - Fischer & Barth. Key competencies: reconciling means and ends in education for sustainable consumption. P.41-60. EXCLUDE ON PAPERS SUBJECT. - Leo & Wickenberg. Under one umbrella: professional norms promoting education for sustainable development at the school level p.61-79. EXCLUDE ON PAPERS SUBJECT. - Madsen et al. Linking health education and sustainability education in schools: local transformations of international policy, p.81-109. EXCLUDE ON PAPERS SUBJECT. - Senior et al. Becoming a health promoting school: using a ‘change agent’ to influence school structure, ethos and ensure sustainability. P.131-153. EXCLUDED ON PAPERS SUBJECT – FOCUSES ON IMPLEMENTATION. - Oddrun & Rowling. Implementation strategies to promote and sustain health and learning in school. P.233-252. EXCLUDED ON EVIDENCE – NOT EMPIRICAL.   Through this page Home - where evidence meets experience > Handbook Sections (HS) > Local Mechanisms in Implementation (HS) – <http://www.schools-for-all.org/page/Local+Mechanisms+in+Implementation+%28HS%29> – identified and screened on title and abstract:  August, G.J., Winters, K.C., Realmuto, G.M., Tarter, R., Perry, C., & Hektner, J.M. (2004). Moving evidence-based drug abuse prevention programs from basic science to practice: Bridging the efficacy-effectiveness interface. *Substance Use & Misuse*, 39(10-12), 2017-2053. EXCLUDE ON PAPERS SUBJECT.  Adelman, H.S., & Taylor, L. (2003). Creating school and community partnerships for substance abuse prevention programs.*The Journal of Primary Prevention*, 23(3). EXCLUDE ON EVIDENCE – NOT EMPIRICAL. Brounstein, P.J., Gardner, S.E., & Backer, T.E. (2006). Research to practice: Efforts to bring effective prevention to every community. *The Journal of Primary Prevention*, 27(1). EXCLUDE ON PAPERS SUBJECT. Petrosino, A. (2003). Standards for Evidence and Evidence for Standards: The Case of School-Based Drug Prevention. *The Annals of the American Academy of Political and Social Science*,*587*(1), 180-207. EXCLUDE ON PAPERS SUBJECT. Rohrbach, L.A., Ringwalt, C.L., Ennett, S.T., & Vincus, A.A. (2005). Factors associated with adoption of evidence-based substance use prevention curricula in US school districts. *Health Education Research*, *20*(5), 514-526. EXCLUDE ON PAPERS SUBJECT.  Han, S., & Weiss, B. (2005). Sustainability of teacher implementation of school-based mental health programs. *Journal of Abnormal Child Psychology*, *33*(6), 665-679. EXCLUDE ON DUPLICATION.  Sobeck, J.L., Abbey, A., & Agius, E. (2006). Lessons learned from implementing school-based substance abuse prevention curriculums. *Children and Schools*, *28*(2), 77-85.EXCLUDE ON PAPERS SUBJECT. Johnson, K., Hays, C., Daley, C., & Hayden Center. (2004). Building capacity and sustainable prevention innovations: A sustainability planning model. *Evaluation and Program Planning*, *27*, 135-149. EXCLUDE ON EVIDENCE – NOT EMPIRICAL.  Berryhill, J.C., & Prinz, R.J. (2003). Environmental interventions to enhance student adjustment: Implications for prevention.*Prevention Science*, *4*(2).  EXCLUDE ON PAPERS SUBJECT. Pentz, M.A., Jasuja, G.K., Rohrbach, L.A., Sussman, S., & Bardo, M.T. (2006). Translation in tobacco and drug abuse prevention research. *Evaluation & the Health Professions*, *29*(2), 246-271.EXCLUDE ON PAPERS SUBJECT. Murnane, A., Snow, P., Farringdon, F., Munro, G., Midford, R., & Rowland, B. (2002). *National school drug education strategy. Effective implementation practice in relation to school drug education*. Perth, Australia: National Drug Research Institute, Curtin University. EXCLUDE ON PAPERS SUBJECT. Swisher, J.D. (2000). Sustainability of prevention. *Addictive Behaviors*, *25*, 965-973. EXCLUDE ON EVIDENCE – NOT EMPIRICAL. Payne, A.A., Gottfredson, D.C., & Gottfredson, G.D. (2006). School predictors of the intensity of implementation of school-based prevention programs: Results from a national study. *Prevention Science*, *7*(2), 225-237. EXCLUDE ON PAPERS SUBJECT.  Ringwalt, C.L., Ennett, S., Johnson, R., Rohrbach, L.A., Simons-Rudolph, A., Vincus, A., & Thorne, J. (2003). Factors associated with fidelity to substance use prevention. Curriculum guides in the nation’s middle schools.*Health Education & Behavior*, *30*(3), 375-391. EXCLUDE ON PAPERS SUBJECT. Baker, P.J. (2006). Developing a blueprint for evidence-based drug prevention in England. *Drugs: Education, Prevention and Policy*, *13*(1), 17-32. EXCLUDE ON PAPERS SUBJECT. Hawthorne, G. (2001). Drug education: Myth and reality. *Drug and Alcohol Review*, *20*(1), 111-119. EXCLUDE ON PAPERS SUBJECT. Poulin, C., & Nicholson, J. (2005). Should harm minimization as an approach to adolescent substance use be embraced by junior and senior high schools? Empirical evidence from an integrated school and community-based demonstration intervention addressing drug use among adolescents. *International Journal of Drug Policy*, *16, EXCLUDE ON PAPERS SUBJECT.* Ennett, S.T., Ringwalt, C. L., Thorne, J., Rohrbach, L.A., Vincus, A., Simons-Rudolph, A., & Jones, S. (2003). A comparison of current practice in school-based substance use prevention programs with meta-analysis findings. *Prevention Science*, *4*(1), 1-14. EXCLUDE ON PAPERS SUBJECT. Bishop, D., Bryant, K.S., Giles, S.M., Hansen, W.B., & Dusenbury, L. (2006). Simplifying the delivery of a prevention program with web-based enhancements. *Journal of Primary Prevention*, *27*(4), 433-444. EXCLUDE ON PAPERS SUBJECT. Roche, A.M. (2002). *Workforce development issues in the AOD field: A briefing paper for the inter-governmental committee on drugs*. Retrieved September 30, 2007, from <http://www.nceta.flinders.edu.au/pdf/issues.pdf> EXCLUDE ON PAPERS SUBJECT. Toumbourou, J.W., Rowland, B., Jefferies, A., Butler, H., & Bond, L. (2004). *Preventing drug-related harm through school re-organisation and behavior management* [Prevention research evaluation report No. 12]. Melbourne, Australia: Australia Drug Foundation. Retrieved September 30, 2007, from <http://www.druginfo.adf.org.au/downloads/Prevention_Research_Quarterly/PRQ_04Nov_Early_intervention_in_schools.pdf> EXCLUDE ON PAPERS SUBJECT. Greenberg, M.T., Weissberg, R.P., O’Brien, M.U., Zins, J.E., Fredricks, L., Resnik, H., et al. (2003). Enhancing school-based prevention and youth development through coordinated social, emotional, and academic learning. *American Psychologist*, *58*, 466–474. EXCLUDE ON PAPERS SUBJECT. Bond, L., Glover, S., Godfrey, C., Butler, H., & Patton, G.C. (2001). Building capacity for system-level change in schools: Lessons from the gatehouse project. *Health Education and Behavior*, *28*(3), 368-383. EXCLUDE ON PAPERS SUBJECT (FOCUSES ON IMPLEMENTATION). Stormshak, E.A., Dishion, T.J., Light, J., & Yasui, M. (2005). Implementing family-centered interventions within the public middle school: Linking service delivery to change in student problem behavior.*Journal of Abnormal Child Psychology*, *33*(6), 723-733. EXCLUDE ON PAPERS SUBJECT. Inchley, J., Muldoon, J., & Currie, C. (2007). Becoming a health promoting school: Evaluating the process of effective implementation in Scotland. *Health Promotion International*, *22*(1), 65-71. EXCLUDE ON PAPERS SUBJECT (FOCUSES ON IMPLEMENTATION) Abrams, D. B., & Clayton, R. R. (2001). Transdisciplinary research to improve brief interventions for addictive behaviors. In P. M. Monti, S. M. Colby & T. A. O'Leary (Eds.), *Adolescents, alcohol, and substance abuse: Reaching teens through brief interventions*. Retrieved September 30, 2007, from <http://ajp.psychiatryonline.org/cgi/reprint/159/11/1958> EXCLUDE ON PAPERS SUBJECT. Midford, R., Wilkes, D., & Young, D. (2005). Evaluation of the in touch training program for the management of alcohol and other drug use issues in schools. *Journal of Drug Education*, *35*(1), 1-14. EXCLUDE ON PAPERS SUBJECT. Berryhill, J.C., & Prinz, R.J. (2003). Environmental interventions to enhance student adjustment: Implications for prevention.*Prevention Science*, *4*(2), 65-87.EXCLUDE ON PAPERS SUBJECT.  Checked references on [Home - where evidence meets experience](http://www.schools-for-all.org/page/Home+-+where+evidence+meets+experience) > [Handbook Sections (HS)](http://www.schools-for-all.org/page/Handbook+Sections+%28HS%29) > Using Evidence-based Implementation Models in School Health, Safety & Social Development (HS) - <http://www.schools-for-all.org/page/Using+Evidence-based+Implementation+Models+in+School+Health%2C+Safety+%26+Social+Development+%28HS%29> but none were new and relevant.  Checked reference on Home - where evidence meets experience > [Bibliographies & Toolboxes (BT)](http://www.schools-for-all.org/page/Bibliographies+%26+Toolboxes+%28BT%29) > [Sun Safety/Skin Cancer Prevention (BT)](http://www.schools-for-all.org/page/Sun+Safety%2FSkin+Cancer+Prevention+%28BT%29).. http://www.schools-for-all.org/page/Sun+Safety%2FSkin+Cancer+Prevention+%28BT%29   - Screened one on abstract Milne E, Jacoby P, Giles-Corti B, Cross D, Johnston R, English DR.(2006) [The impact of the kidskin sun protection intervention on summer suntan and reported sun exposure: was it sustained?](http://www.ncbi.nlm.nih.gov/pubmed/16330090?ordinalpos=1&itool=EntrezSystem2.PEntrez.Pubmed.Pubmed_ResultsPanel.Pubmed_DiscoveryPanel.Pubmed_Discovery_RA&linkpos=3&log$=relatedarticles&logdbfrom=pubmed) Prev Med. Jan;42(1):14-20. Epub 2005 Dec 5.   EXCLUDED – FOCUSED ON YPS OUTCOMES.  Checked references [Home - where evidence meets experience](http://www.schools-for-all.org/page/Home+-+where+evidence+meets+experience) > [Handbook Sections (HS)](http://www.schools-for-all.org/page/Handbook+Sections+%28HS%29) > Ministry, Agency/School Board, School & Professional Capacity in Mental Health Promotion (HS) on http://www.schools-for-all.org/page/Ministry%2C+Agency%2FSchool+Board%2C+School+%26+Professional+Capacity+in+Mental+Health+Promotion+%28HS%29   - Darcy A. Santor, Alexa L. Bagnell (2012) [Maximizing the Uptake and Sustainability of School-Based Mental Health Programs: Commercializing Knowledge](http://www.childpsych.theclinics.com/issues), Child and Adolescent Psychiatric Clinics of North America Volume 21, Issue 1, 81-92. EXCLUDE ON DUPLICATION – FOUND IN DATABASE SEARCH. - Kari M. Gloppen, Michael W. Arthur, J. David Hawkins, Valerie B. Shapiro (2012) [Sustainability of the Communities That Care Prevention System by Coalitions Participating in the Community Youth Development Study](http://www.jahonline.org/article/S1054-139X%2811%2900698-7/abstract) Journal of Adolescent Health (In Press) EXCLUDE ON INTERVENTION – NOT SCHOOL-BASED - Russell L Gruen, Julian H Elliott, Monica L Nolan, Paul D Lawton, et al. (2008) [Sustainability science: an integrated approach for health-programme planning](http://www.thelancet.com/journals/lancet/article/PIIS0140-6736%2808%2961659-1/abstract)The Lancet Vol. 372, Issue 9649, Pages 1579-1589 EXCLUDED –NOT EMPIRICAL - Mark E. Feinberg, Daniel E. Bontempo, Mark T. Greenberg (2008) [Predictors and Level of Sustainability of Community Prevention Coalitions](http://www.ajpmonline.org/article/S0749-3797%2808%2900237-7/abstract) American Journal of Preventive Medicine Vol. 34, Issue 6, Pages 495-501 EXCLUDED ON INTERVENTION – NOT SCHOOL-BASED - Kurt C Stange, Meredith A Goodwin, Stephen J Zyzanski, Allen J Dietrich [Sustainability of a practice-individualized preventive service delivery intervention](http://www.ajpmonline.org/article/S0749-3797%2803%2900219-8/abstract)American Journal of Preventive Medicine Vol. 25, Issue 4, Pages 296-300 EXCLUDE ON INTERVENTION – NOT SCHOOL BASED. | One include on T&A but excluded on full text. |
| International Union for Health Promotion and Education (www.iuhpe.org) | 4/07/18 | Searched the ‘School health’ section of IUHPE thematic resources in ‘Publications’ tab - <https://www.iuhpe.org/index.php/en/iuhpe-thematic-resources/298-on-school-health>  Looked at the book by Aldinger et al (eds) (2009) “Case studies in global school health promotion”. EXCLUDED AS NOT EMPIRICAL.  Searched the ‘research’ section of IUHPE thematic resources in ‘Publications’ tab. | None. |
| National Centre for Social Research (www.natcen.ac.uk/our-research/) | 16/07/18 | Searched all publications under the following sections:  “Children & young people”– NONE RELEVANT ON TITLE AND ABSTRACT.  “Health & wellbeing” – NONE RELEVANT ON TITLE AND ABSTRACT.  “Schools, education & training”. – NONE RELEVANT ON TITLE AND ABSTRACT. | None. |
| National Foundation for Education Research (https://www.nfer.ac.uk/) | 16/7/18 | Looked under “Publications & research” and the following categories:  “Health and wellbeing”, “ Leadership and management in schools”, “Other curriculum subjects”, “Professional development”, “Teaching and pedagogy”, “Teaching and innovation” – NONE RELEVANT ON TITLE AND ABSTRACT | None. |
| NHS Evidence Library  (https://www.evidence.nhs.uk) | 18/7/18 | Searched using the standard search terms and filtered results by selecting “Primary research” and “Ongoing trials” categories. 493 records identified. Screened on title.  X screened on abstract:  Wyatt et al (2018). Cluster randomised controlled trial and economic and process evaluation to determine the effectiveness and cost effectiveness of a novel intervention [Healthy Lifestyles Programme (HeLP)] to prevent obesity in school children. EXCLUDE ON PAPER’S SUBJECT. LOOKS AT YPS OUTCOMES. | None. |
| NHS Health Scotland library  (http://www.healthscotland.scot/) | 18/7/18 | Searched the “Publications” section of the website. The search engine brings up pre-specified key words to search under. Searched the following:   - Sustain, sustainable, sustained, sustainability – no reports. - Continued – no reports. - Maintain or maintained – 2 reports, neither relevant. - Embed – no reports. - Integration – 1 report, not relevant. - Discontinued – 1 report, not relevant. - Mainstreaming – 3 reports, none relevant. - Persistence – 1 report, not relevant. | None. |
| School Health Education Unit (www.sheu.org.uk) | 18/7/18 | Searched the “Publications” section of the website. The search engine does not allow Boolean terms.  Education and Health Journal Archive – searched using the following search terms:   - Sustain – 1 report identified, screened on full text: Grant,S 2005. Tipu Ka Rea/ to grow, expand and multiply: an operational model for developing sustainable health-promoting schools in Aotearoa/New Zealand. *Education and Health***23**(3),44-46. EXCLUDED ON EVIDENCE – NOT EMPIRICAL. - Sustainability/sustained – no reports. - Continued/continuation – no reports. - Maintenance/maintained/maintain – 1 report, excluded on title. - Institutionalisation/institutionalization – no reports. - Routinisation/routinization – no reports. - Embed/embedded – no reports. - Incorporation – no reports. - Integration – no reports. - Normalisation/normalization – no reports. - Stabilization – no reports. - Durability – no reports. - Long-term implementation/long term implementation – no reports. - Discontinuation/discontinued – no reports. - Mainstreaming – no reports. - Scale up/scale-up/scaling up/scaling-up – no reports. - Endurance – no reports. - Persistence – no reports. |  |
| Schools for Health in Europe (http://www.schools-for-health.eu/she-network) | 18/7/18 | Searched under “Resources”, no relevant reports found.  Searched under “Publications and reports” under “Research Group”.  One book identified, “Schools for Health and Sustainability: Theory, research and Practice” was EXCLUDED ON DUPLICATION as identified through another website, the International School Health Network.  Two journals promoted Health Promotion International and Health Education, both of which already included in the database search. |  |
| WHO  (http://www.who.int/) | 19/7/18 | Searched the WHO library catalogue. http://kohahq.searo.who.int/ using the standard search terms, filtered by date range 1996-2018 and English only. 255 records. Screened on title. | None. |
