# Supplementary material for: The sustainability of public health interventions in schools: a systematic review
Source: Implement Sci. 2020 Jan 6;15:4. doi: 10.1186/s13012-019-0961-8 (PMC6945701; doi:10.1186/s13012-019-0961-8)
Supplement: Supplementary file 3 — Additional file 3: Contact with subject experts. [file 13012_2019_961_MOESM3_ESM.docx]

**Additional file 3: Contact with subject experts**

A number of experts were contacted for this review by email on 24^th^ July 2018. The names of the experts contacted and their institutions, and template of the email sent are detailed below.

| Alyssa Lederer | Assistant Professor, Tulane University, Louisiana, USA |
| --- | --- |
| Bahr Weiss | Associate Professor of Psychology and Human Development, Vanderbilt University, Nashville, USA |
| Brian Flay | Professor, School of Social and Behavioral Health Sciences, Oregon State University, USA |
| Bruce Taylor | Senior Fellow, Public Health, University of Chicago, USA |
| Carl May | Professor, University of Southampton, UK |
| Carolyn C. Johnson | Endowed Professor, School of Public Health and Tropical Medicine, Tulane University, Louisiana, USA |
| Cate Egan | Assistant Professor, University of Idaho, USA |
| Claire V. Crooks | Associate Professor, Western Education, USA |
| Collin Webster | Associate Dean for Research and Innovation Professor, College of Education, University of South Carolina, USA |
| Danny Wight | Professor, Institute of Health & Wellbeing, University of Glasgow, UK |
| David Foxcroft | Professor of Community Psychology and Public Health, Oxford Brooks, UK |
| Deanna M. Hoelscher | Director, Michael & Susan Dell Center for Healthy Living, USA |
| Douglas Luke | Director, Center for Public Health Systems Science, Washington University in St Louis, USA |
| Elling Bere | Professor, Universitetet Agder, Norway |
| Emily Ozer | Professor, Community Health Sciences, University of California, Berkeley, USA |
| Erum Nadeem | Assistant Professor, Department of Child and Adolescent Psychiatry, New York University, USA |
| George Patton | Professor of Population Health Studies of Adolescents, University of Melbourne, Australia |
| Graham Moore | Senior Lecturer, School of Social Sciences, University of Cardiff, UK |
| Helen Weiss | Professsor of Epidemiology and Director of the MRC Tropical Epidemiology Group, LSHTM, UK |
| Honor Young | Lecturer in quantitative research methods, School of Social Sciences, University of Cardiff, UK |
| J. David Hawkins | Endowed Professor in Prevention, University of Washington, USA |
| Jeremy Segrott | Lecturer, University of Cardiff, UK |
| John E. Lochman | Professor and Doddridge Saxon Chair in Clinical Psychology, University of Alabama, USA |
| John P. Elder | Distinguished Professor, San Diego State University, USA |
| John Santelli | Professor, Population and Family Health and Pediatrics, University of Columbia, USA |
| Judi Kidger | Senior Research Fellow in Public Health, University of Bristol, UK |
| Judy Hutchings | Professor in Psychology, University of Bangor, UK |
| Karen Devries | Associate Professor in Social Epidemiology, LSHTM, UK |
| Karen E. Schetzina | Department of Pediatrics, East Tennessee State University, USA |
| Karin Coyle | Chief Science Officer, ETR Associates, USA |
| Kimberly J. Rauscher | Associate Professor, West Virginia University, USA |
| Larry Aber | Willner Family Professor of Psychology and Public Policy, NYU Steinhardt, USA |
| Laurence Moore | Professor, Director of the MRC/CSO Social and Public Health Sciences Unit, University of Glasgow, UK |
| Leslie A. Lytle | Professor, Department of Health Behavior, Gillings School of Global Public Health, USA |
| Lyndal Bond | Professor of Population Health & Evaluation, Victoria University, Melbourne, Australia |
| Marion Henderson | Senior Investigator Scientist, MRC/CSO Social and Public Health Sciences Unit, University of Glasgow, UK |
| Mark Greenberg | Edna Bennett Chair and Professor, Human Development & Psychology, Penn State, USA |
| Maurice J Elias | Professor, Rutgers School of Arts and Sciences, USA |
| Neil Humphrey | Professor of Psychology of Education, University of Manchester, UK |
| Penelope Hawe | Professor, University of Sydney, Australia |
| Pierre Pluye | Professor, Department of Family Medicine, McGill University, USA |
| Rebecca Muckelbauer | Berlin School of Public Health, Charite University Medical Center Berlin |
| Ricardo Catalano | Professor, University of Washington, USA |
| Rona Campbell | Professor of Public Health Research, University of Bristol, UK |
| Russell E Glasgow | Professor, University of Colorado Denver, USA |
| Sachin Shinde | LSHTM, UK |
| Sarah Friend | Evaluation Director, School of Nursing, University of Minnesota, USA |
| Sheldon L. Loman | Professor, Graduate School of Education, Portland State University, USA |
| Stavroula K. Osganian | Co-Chief, Clinical Research Center, Boston Children’s Hospital, USA |
| Steven H. Kelder | Distinguished Professor in Spirituality and Healing, Michael & Susan Dell Center for Healthy Living, USA |
| Suzanne Audrey | Senior Research Fellow, University of Bristol, UK |
| Tamsin Ford | Professor of Child and Adolescent Psychiatry, University of Exeter, UK |
| Tan Leng Goh | Assistant Professor of Physical Education & Human Performance  Central Connecticut State University, USA |
| Tena L. St Pierre | Penn State, USA |
| Thomas L. McKenzie | Professor Emeritus, School of Exercise and Nutritional Sciences, San Diego State University, USA |
| Trish Gorely | Senior Lecturer, University of Stirling, UK |
| Vikram Patel | The Pershing Square Professor of Global Health, Harvard University, USA |
| NB: email delivery failed to Susan S. Han, Marieke Dijkman and Marthe Deschesnes. No alternative contact addresses could be found. | |

**Email sent to experts**

Dear all,

I am a researcher from the London School of Hygiene & Tropical Medicine (<https://www.lshtm.ac.uk/>)

We are currently undertaking a systematic review entitled ***“*The barriers and facilitators to sustaining public health interventions in schools in OECD countries***”.*Please find the protocol here: <https://www.crd.york.ac.uk/prospero/display_record.php?RecordID=76320>

I am writing to you today as an expert in the implementation and sustainability of health-related interventions in schools. I would like you to inform me of **any research of which you are aware that may be relevant to this review**. The table below summarises the types of study in which we are interested.

| **Definition of sustainability for the review** | **Studies focused on the continuation or discontinuation of a school-based public health intervention once external funding/resources have come to an end.** |
| --- | --- |
| Participants | Delivered to children aged 5 to 18 years and conducted in an OECD country. |
| Intervention | Delivered partly or wholly within school hours. Included health outcomes among its primary outcomes.  Universal or targeted approach, encompassing one of more of the following:  A formal health curriculum, Ethos or environment of the school Engagement with families or communities or both |
| Outcomes targeted by the intervention | Obesity or overweight or body size, physical activity or sedentary behaviours, nutrition, tobacco use, alcohol use, other drug use, sexual health, mental health or  emotional well-being, bullying, infectious diseases, safety or accident prevention, body image or eating disorders, skin or sun safety, oral |
| Study design | Empirical - qualitative or quantitative. Fieldwork **must** have been carried out after original external funding/resources to adopt and implement the intervention had ended. |

At the end of this email is a list of relevant studies of which we are already aware (of which many of you are the authors).

Ideally I would be very grateful if you could let me know of additional relevant studies by email by 20th August 2018. However, if this is not possible, please could you indicate if and by when you would be able to respond?

If there are other experts you would recommend we contact, please do let me know.

If you have any questions, please do not hesitate to get in touch.

Thank you in advance for your assistance on this matter.

Best regards,

Lauren Herlitz
