# Supplementary material for: The sustainability of public health interventions in schools: a systematic review
Source: Implement Sci. 2020 Jan 6;15:4. doi: 10.1186/s13012-019-0961-8 (PMC6945701; doi:10.1186/s13012-019-0961-8)
Supplement: Supplementary file 4 — Additional file 4: Data extraction and quality appraisal form. [file 13012_2019_961_MOESM4_ESM.docx]

**Additional file 4: Data extraction and quality appraisal form**

| *Consider if the study has reported the following. Provide justifications for your response wherever necessary.*  *If not reported, write ‘Not stated’. If not applicable, write ‘NA’. Do not leave blank.* | | | | |
| --- | --- | --- | --- | --- |
| Completed by: |  | Date: | |  |
| 1. **IDENTIFICATION OF DOCUMENT OR ARTICLE** | | | | |
| 1.1 Citation | | |  | |
| 1. **STUDENT CHARACTERISTICS - STUDY SAMPLE FOR TRIAL PHASE** | | | | |
| 2.1 Age/grades | | |  | |
| 2.2 Sex (*boys, girls, mixed*) | | |  | |
| 2.3 SES | | |  | |
| 2.4 Ethnicity | | |  | |
| 1. **SCHOOLS - STUDY POPULATION FOR TRIAL PHASE** | | | | |
| 3.1 Education phase (*Primary/Elementary 5-10 yrs; Secondary/Middle School 11-13 yrs or High School 14-18 yrs*) | | |  | |
| 3.2 School type (*State; Private; Not stated*) | | |  | |
| 3.3 Single sex/mixed sex | | |  | |
| 3.4 Geographic location (*Country; area(s) of country*) | | |  | |
| 1. **DESCRIPTION OF THE INTERVENTION** | | | | |
| 4.1 NAME: Intervention name (*explain any abbreviations*) | | |  | |
| 4.2 AIM: Overall aim(s) of the intervention | | |  | |
| 4.3 TARGETED or UNIVERSAL? | | |  | |
| 4.4 MATERIALS: Describe any physical or informational materials used in the intervention, including those provided to participants or used in intervention delivery or in training of intervention provider | | |  | |
| 4.5 METHOD AND MODE: Describe each of the procedures, activities, and/or processes used in the intervention, and include the mode of delivery (e.g. face-to-face, internet) and whether it was provided individually or in a group. | | |  | |
| 4.6 PROVIDER: Person(s) providing the intervention (*e.g. teachers, counsellors, external facilitators*). For each type of provider, describe whether they were external to the school, their expertise, background and any specific training given. | | |  | |
| 4.7 LOCATION: Describe the location of the intervention (e.g. classroom, playground) | | |  | |
| 4.8 DOSE AND SCHEDULE: Describe the number of times the intervention was delivered an over what period of time and include the number of sessions, their schedule, and their duration | | |  | |
| 4.9 TAILORING: If the intervention was intended to be adaptable, describe how. | | |  | |
| 4.10 MODIFICATIONS: If the intervention was modified during the course of the study, describe the changes | | |  | |
| 1. **KEY DATES** | | | | |
| - 1. Trial phase baseline evaluation data collection date (MM/YYYY) | | |  | |
| - 1. Start of intervention trial phase implementation (MM/YYYY) | | |  | |
| - 1. End of intervention trial phase implementation (MM/YYYY) (i.e. externally funded materials or providers are no longer given to schools. Provide details of any uncertainty) | | |  | |
| - 1. Trial phase post-intervention evaluation data collection date (MM/YYYY) | | |  | |
| - 1. Sustainability phase evaluation data collection date(s) (MM/YYYY) (nb, may be called follow-up data collection) | | |  | |
| 1. **TRIAL PHASE - STUDY DESIGN/METHODOLOGY** | | | | |
| 6.1 Citation for the effectiveness evaluation | | |  | |
| 6.2 Overall study design (e.g RCT; matched comparison; uncontrolled before/after; cross-sectional) | | |  | |
| 6.3 Number of schools (*state how many were intervention and control*) | | |  | |
| 6.4 Number of student participants (*state how many were intervention and control*) | | |  | |
| 6.5 Any indication from this evaluation of significant effects on primary and secondary outcomes post-intervention? | | |  | |
| 6.6 Any details about how successfully the intervention was implemented | | |  | |
| 6.7 Was the study of sustainability a long-term follow-up for this effectiveness evaluation? [*Yes/No*] | | |  | |
| 1. **SUSTAINABILITY PHASE – STUDY DESIGN/METHODOLOGY** | | | | |
| 7.1 Research questions or hypotheses | | |  | |
| 7.2 Definition of sustainability used (if there is one) | | |  | |
| 7.3 Rationale for the sustainability of the intervention (*include name of conceptual framework/model if used*) | | |  | |
| 7.4 Overall study design | | |  | |
| 7.5 Describe what schools were selected for study and rationale | | |  | |
| 7.6 School sample size, response rate, and characteristics | | |  | |
| 7.7 Describe what practitioner/stakeholder participants were selected for study and rationale (*if applicable*) | | |  | |
| 7.8 Practitioner participant sample size, response rate, and characteristics. | | |  | |
| 7.9 Describe what student participants were selected for study and rationale (*if applicable*) | | |  | |
| 7.10 Student participant sample size, response rate, and characteristics (*if applicable*). | | |  | |
| 7.11 Methods of data collection (*when was data collected, from whom, what data collection method was used)* | | |  | |
| 7.12 Methods of data analysis (*include unit of analysis*) | | |  | |
| 1. **SUSTAINABILITY STUDY FINDINGS** | | | | |
| 8.1 Give details on how many schools sustained the intervention or various components of the intervention (if relevant to study).  *Summarise in a format similar to:*   - *X/Y (%) intervention schools sustained Z component.* - *X/Y (%) control schools sustained Z component.* | | |  | |
| 8.2 Give details of sustained health outcomes for students (if applicable).  *Summarise* | | |  | |
| 8.3 Provide any results which examine the relationship between intervention (dis)continuation and outcome sustainability (if applicable)  *Summarise* | | |  | |
| 8.4 Give details of all results relating to barriers and facilitators of sustainability.  *Verbatim (include page numbers).* | | |  | |
| 8.5 Give details of any adaptations to the intervention after intervention period.  *Verbatim (include page numbers)* | | |  | |
| 8.6 Additional insights from the authors’ discussion section.  *Verbatim (include page numbers).* | | |  | |
| 1. **QUALITY APPRAISAL** | | | | |
| *C1: Justification*: Was there an explicit account of the theoretical framework and/or inclusion of a literature review? Did the report provide an explanation of, and justification for, the focus of the study and the methods used? [No/Partial/Yes] | | |  | |
| *C2: Clearly stated aims/objectives*: Did the report explicitly and clearly state the aims of the study? [No/Partial/Yes] | | |  | |
| *C3: Clear description of context*: Did the report adequately describe the specific circumstances under which the research was developed, carried out and completed? [No/Partial/Yes] | | |  | |
| *C4: Clear description of sample*: Did the report provide adequate details of the sample including details of sampling and recruitment? [No/Partial/Yes] | | |  | |
| *C5: Clear description of methodology*: Did the report provide an adequate description of the methods used to collect and analyse the data? [No/Partial/Yes] | | |  | |
| *C6: Establishing reliability and validity of the data*: Have the researcher demonstrated some attempt to assess the reliability and validity of the data? [No/Partial/Yes] | | |  | |
| *C7: Inclusion of original data*: Did the report present sufficient data in the form of, for example, data tables, direct quotations from interviews or focus groups, or data from observations, to enable the reader to see that the results and conclusions were grounded in the data? [No/Partial/Yes] | | |  | |
| W1: How reliable or trustworthy overall are the findings? (i.e. the extent to which the methods employed were rigorous/could minimise bias and error in the findings) [Low/Medium/High] plus free text explaining the decision | | |  | |
| W2: How useful, overall, were the findings for shedding light on factors relating to the research questions for this review? [Low/Medium/High] plus free text explaining the decision | | |  | |
