# Supplementary material for: The sustainability of public health interventions in schools: a systematic review
Source: Implement Sci. 2020 Jan 6;15:4. doi: 10.1186/s13012-019-0961-8 (PMC6945701; doi:10.1186/s13012-019-0961-8)
Supplement: Supplementary file 5 — Additional file 5: Quality appraisal guidance and ratings. [file 13012_2019_961_MOESM5_ESM.docx]

**Additional file 5: Quality appraisal guidance and ratings**

Guidance on determining reliability (weight of evidence 1)

- For a judgement of ‘high’ reliability, studies need to have taken steps to ensure rigour – a clear ‘Yes’ – in at least five of the seven criteria.
- For a judgement of ‘medium’ reliability, studies will have been rated as ‘Partial’ or ‘Yes’ in at least four of the seven criteria.
- All others should be judged as ‘low’ reliability.

Guidance on determining usefulness (weight of evidence 2)

- For a judgement of ‘high’ on usefulness, studies need to have described, with both breadth and depth, the factors affecting the sustainability or discontinuation of the intervention, and will have privileged the perspectives of participants.
- For a judgement of ‘medium’ on usefulness, studies need to have described, with some breadth and/or depth, the factors affecting the sustainability or discontinuation of the intervention, and will have partially drawn on the views and experiences of participants.
- ‘Low’ usefulness will have some findings of interest regarding the intervention’s sustainability with a minimal amount of depth and will minimally have privileged the views of participants, if at all.

Criteria 1 – 7 rated No, Partial or Yes; Weight of evidence 1 and 2 rated Low, Medium or High.

| Study # | Intervention;  author(s) and year; | C1:  Justification | C2:  Clearly stated aims/  objectives | C3:  Clear description of context | C4:  Clear description of sample | C5:  Clear description of method-ology | C6:  Establish-ing reliability and validity of the data | C7: Inclusion of original data | Weight of evidence 1: Reliability | Weight of evidence 2: Relevance |
| --- | --- | --- | --- | --- | --- | --- | --- | --- | --- | --- |
| 1 | *Project Salsa;*  Elder et al. 1998 | Partial | Yes | No | No | No | No | No | Low | Low |
| 2 | *Adolescent Suicide Awareness Program (ASAP)*;  Kalafat and Ryerson 1999 | Partial | Yes | Yes | No | Partial | No | Partial | Low | Medium |
| 3 | *Child and Adolescent Trial for Cardiovascular Health (CATCH) – health education curriculum*;  Johnson et al. 2003 | Partial | Partial | Yes | Yes | Yes | Partial | Yes | High | Low |
| 4 | *CATCH – PE component*;  Kelder et al. 2003 | Partial | Yes | Yes | Yes | Yes | Partial | Partial | Medium | Medium |
| 5 | *CATCH – all components*;  Lytle et al. 2003 | Partial | Yes | Partial | Yes | Yes | Yes | Yes | Medium | High |
| 6 | *CATCH – PE component*;  McKenzie et al. 2003 | Yes | Yes | Yes | No | No | Partial | Partial | Low | Low |
| 7 | *CATCH – food service component*;  Osganian et al. 2003 | Partial | Yes | Yes | Partial | Yes | Yes | Yes | High | Medium |
| 8 | *CATCH – school climate*;  Parcel et al. 2003 | Partial | Yes | Yes | Partial | Yes | Yes | Yes | High | Low |
| 9 | *CATCH – all components*;  Hoelscher 2004 | Yes | Yes | Yes | Yes | Yes | Yes | Yes | High | Low |
| 10 | *Project ALERT*;  St Pierre and Kaltreider 2004 | Partial | Yes | Yes | Partial | Partial | Partial | No | Low | Low |
| 11 | *School Fruit Programme and the Fruit and Vegetables Make the Marks (FVMM)*;  Bere 2006 | No | Yes | Yes | Partial | Yes | Yes | Yes | High | Low |
| 12 | *Untitled - intervention focused on water consumption*;  Muckelbauer, Libuda, Clausen, and Kersting 2009 | Partial | Yes | Yes | Partial | Partial | Partial | Yes | Medium | Low |
| 13 | *European Network of Health-Promoting Schools*;  Tjomsland et al. 2009 | Partial | Yes | Partial | Partial | Yes | Partial | Yes | Medium | High |
| 14 | *First Step to Success (FSS)*;  Loman, Rodriguez, and Horner 2010 | Partial | Yes | Yes | Partial | Partial | Partial | Partial | Low | Low |
| 15 | *GreatFun2Run*;  Gorely et al. 2011 | Yes | Yes | Partial | Partial | Yes | Yes | Yes | High | Medium |
| 16 | *Winning with Wellness*;  Schetzina et al. 2009 | Partial | Yes | Yes | Partial | Yes | Partial | Yes | Medium | Low |
| 17 | *Fourth R program*;  Crooks et al. 2013 | Partial | Yes | Yes | Partial | Partial | No | Yes | Low | Medium |
| 18 | *New Moves*;  Friend et al. 2014 | Partial | Yes | Yes | Partial | Partial | Partial | No | Medium | Medium |
| 19 | *Youth@work: Talking Safety*;  Rauscher et al. 2015 | Partial | Yes | Yes | Yes | Yes | Partial | Yes | Low | Low |
| 20 | *Cognitive Behavioral Intervention for Trauma in Schools (CBITS)*;  Nadeem and Ringle 2016 | Yes | Yes | Yes | Yes | Yes | Yes | Yes | High | High |
| 21 | *Good Behavior Game (GBG)*;  Dijkman et al. 2017 | Yes | Yes | Partial | Partial | Yes | Partial | Yes | Medium | High |
| 22 | *TAKE 10!*;  Goh et al. 2017 | Yes | Yes | Yes | Partial | Partial | Partial | Yes | Medium | Medium |
| 23 | *School outdoor smoking ban*;  Rozema et al. 2018 | Partial | Yes | Yes | Partial | Partial | Partial | Partial | Low | Medium |
| 24 | *Health Optimizing PE (HOPE)*;  Egan et al. 2019 | Yes | Yes | Yes | Yes | Yes | Yes | Yes | High | Medium |
