# Supplementary material for: The sustainability of public health interventions in schools: a systematic review
Source: Implement Sci. 2020 Jan 6;15:4. doi: 10.1186/s13012-019-0961-8 (PMC6945701; doi:10.1186/s13012-019-0961-8)
Supplement: Supplementary file 7 — Additional file 7: Additional details on sustainability study design participants. [file 13012_2019_961_MOESM7_ESM.docx]

**Additional file 6: Additional details on sustainability study design participants**

| **Study #** | **Intervention;**  **author(s) and year** | **No. of former intervention (FI) schools; response rate** | **No. of schools in comparison group (CG); response rate** | **No. of school personnel; response rate** | **No. other participants or observations** |
| --- | --- | --- | --- | --- | --- |
| 1 | *Project Salsa*;  Elder et al., 1998 | 6 schools;  100% (implied) | N/A | Not known, no details on school-level participants. | N/A |
| 2 | *Adolescent Suicide Awareness Program (ASAP)*;  Kalafat and Ryerson, 1999 | 24 schools;  73% | 7 schools with another youth suicide prevention programme;  54% | 24 staff from FI schools, 11 of whom participated in structured interviews;  100%.  7 staff from CG schools;  100% | N/A |
| 3 | *Child and Adolescent Trial for Cardiovascular Health (CATCH) – health education curriculum*;  Johnson et al., 2003 | 56 schools;  100% | 20 schools (CG1) who received a lower dose of CATCH at the end of the trial, and 12 schools (CG2) who did not receive the intervention;  100%. | 572 teachers from FI schools;  94%  191 teachers from CG1 schools;  90%  127 teachers from CG2 schools;  93% | N/A |
| 4 | *CATCH – PE component*;  Kelder et al., 2003 | 56 schools;  100% | 20 schools (CG1) who received a lower dose of CATCH at the end of the trial, and 12 schools (CG2) who did not receive the intervention;  100%. | 613 staff from FI schools – teachers & PE specialists;  207 staff from CG1 schools;  138 staff from CG2 schools;  Exact response rates not known but between 94 and 100% | *Lesson observations*  645 PE lessons observed (401 from FI schools, 153 from FC schools, and 91 from comparison schools). |
| 5 | *CATCH – all intervention components*;  Lytle et al., 2003 | 56 schools;  100% | 20 schools (CG1) who received a lower dose of CATCH at the end of the trial;  100%. | 160 staff – teachers, PE specialists, food service staff;  91% | *School district personnel*  20 school district administrators |
| 6 | *CATCH – PE component*;  McKenzie et al., 2003 | 56 schools;  100% | 20 schools (CG1) who received a lower dose of CATCH at the end of the trial;  100%. | 613 staff from FI schools – teachers & PE specialists;  207 staff from CG1 schools;  Exact response rates not known but between 94 and 100% | *Lesson observations*  554 PE lessons observed (401 from FI schools and 153 from FC schools) |
| 7 | *CATCH – food service component*;  Osganian et al., 2003 | 56 schools;  100% | 20 schools (CG1) who received a lower dose of CATCH at the end of the trial;  100%. | 203 FI cooks/ technicians;  94%  82 FC cooks/  technicians;  99%  . |  |
| 8 | *CATCH – school climate*;  Parcel et al., 2003 | 56 schools;  100% | Not applicable | 613 staff from FI schools – teachers & PE specialists;  Exact response rates not known but between 94 and 100% | *Lesson observations* 401 PE lessons observed |
| 9 | *CATCH – all intervention components*;  Hoelscher et al., 2004 | 56 schools;  100% | 20 schools (CG1) who received a lower dose of CATCH at the end of the trial, and 12 schools (CG2) who did not receive the intervention;  100%. | 613 staff from FI schools – teachers & PE specialists;  207 staff from CG1 schools;  138 staff from CG2 schools;  Exact response rates not known but between 94 and 100%  . | *Lesson observations*  645 PE lessons observed (401 from FI schools, 153 from FC schools, and 91 from comparison schools). |
| 10 | *Project ALERT*;  St Pierre and Kaltreider, 2004 | 8 schools;  100% | Not applicable | Not known |  |
| 11 | *School Fruit Programme and the Fruit and Vegetables Make the Marks (FVMM)*;  Bere, 2006 | 9 schools;  100% | 10 schools;  100%. | Not applicable | *Students*  577 students at baseline, 517 students (286 FI and 231 FC) post-trial phase and one year post-trial phase. |
| 12 | *Untitled - intervention focused on water consumption*;  Muckelbauer et al., 2009 | 17 schools;  100% | Not applicable | 11 head teachers;  100% |  |
| 13 | *European Network of Health-Promoting Schools*;  Tjomsland et al., 2009 | 7 schools;  70% | Not applicable | 7 head teachers;  100% |  |
| 14 | *Winning with Wellness*;  Schetzina et al., 2009 | 1 school;  100% | Not applicable | 29 teachers;  98% | N/A |
| 15 | *First Step to Success*;  Loman et al., 2010 | 29 schools;  13/29 school districts (45%) had continued to use the intervention. District administrators nominated schools. | Not applicable | 29 staff – head teachers, teachers, counsellors, psychologist, speech-language therapist, coach;  100% |  |
| 16 | *GreatFun2Run*;  Gorely et al., 2011 | 4 schools;  100% | Not applicable | 8 teachers;  unknown | *Longitudinal data on students’ outcomes*  4 FI schools, 4 FC schools, and 8 secondary schools (approx. a third of students had moved on to secondary school).  *Students*  589 students at baseline, 507 students post-trial phase, 421 students 20 months post-trial phase (206 FI and 215 FC) – outcome data.  72 FI students – focus groups on views and experiences. |
| 17 | *Fourth R program*; Crooks et al., 2013 | Not applicable | Not applicable | 197 teachers;  47% | N/A |
| 18 | *New Moves*;  Friend et al. 2014 | 6 schools;  100% | 6 schools;  100%  Teachers from CG school received a lower dose of New Moves at the end of the trial. | 5 teachers from FI schools;  100%  5 teachers from CG schools;  100% | *Lesson observation*  10 PE lessons (one per school). |
| 19 | *Youth@work: Talking Safety*;  Rauscher et al., 2015 | Not applicable | Not applicable | 104 teachers;  45% | N/A |
| 20 | *Cognitive Behavioral Intervention for Trauma in Schools (CBITS)*;  Nadeem and Ringle, 2016 | Not known | Not applicable | 14 clinicians;  70% | *School district personnel*  2 school district staff. |
| 21 | *Good Behavior Game*;  Dijkman et al., 2017 | 16 schools;  94% | Not applicable | 16 teachers/  GBG co-ordinators;  94% | N/A |
| 22 | *TAKE 10!*  Goh et al., 2017 | 2 schools;  Opportunity sample | Not applicable | 15 teachers;  Not known | N/A |
| 23 | *School outdoor smoking ban*;  Rozema et al., 2018 | 438 schools;  Not known – 919 schools, of which 438 currently had the intervention, | Not applicable | 438 head teachers;  100%.  A sub-sample of 15 participated in interviews. | N/A |
| 24 | *Health Optimizing PE (HOPE)*;  Egan et al., 2019 | 1 school;  100% | Not applicable | 7 teachers;  100% | *Students*  5 students, focus group.  *Research team*  5 research team members, interviews |

*Estimated as the time between the last year of the trial phase evaluation and the last year of the sustainability phase evaluation.
